# A Novel Tau Antibody Detecting the First Amino-Terminal Insert Reveals Conformational Differences Among Tau Isoforms

**DOI:** 10.3389/fmolb.2020.00048

**Published:** 2020-03-31

**Authors:** Joke Verelst, Nick Geukens, Sabiha Eddarkaoui, Dorien Vliegen, Elien De Smidt, Joëlle Rosseels, Vanessa Franssens, Sofie Molenberghs, Cindy Francois, Erik Stoops, Maria Bjerke, Sebastiaan Engelborghs, Mohamed Laghmouchi, Sofie Carmans, Luc Buée, Eugeen Vanmechelen, Joris Winderickx, Debby Thomas

**Affiliations:** ^1^Functional Biology, KU Leuven, Heverlee, Belgium; ^2^PharmAbs, KU Leuven, Leuven, Belgium; ^3^Univ. Lille, Inserm, CHU-Lille, UMRS1172, Lille Neuroscience & Cognition, LabEx DISTALZ, Alzheimer & Tauopathies, Lille, France; ^4^ADx NeuroSciences NV, Ghent, Belgium; ^5^Reference Center for Biological Markers of Dementia (BIODEM), Institute Born-Bunge, University of Antwerp, Wilrijk, Belgium; ^6^Department of Neurology and Center for Neurosciences, UZ Brussel and Vrije Universtieit Brussel (VUB), Brussels, Belgium; ^7^reMYND NV, Bio-Incubator, Heverlee, Belgium

**Keywords:** yeast, *Saccharomyces cerevisiae*, Tau, Tau isoforms, monoclonal antibodies, conformational differences

## Abstract

As human Tau undergoes pathologically relevant post-translational modifications when expressed in yeast, the use of humanized yeast models for the generation of novel Tau monoclonal antibodies has previously been proven to be successful. In this study, human Tau2N4R-ΔK280 purified from yeast was used for the immunization of mice and subsequent selection of high affinity Tau-specific monoclonal antibodies. The characterization of four novel antibodies in different Tau model systems yielded a phosphorylation-dependent antibody (15A10), an antibody directed to the first microtubule-binding repeat domain (16B12), a carboxy-terminal antibody (20G10) and an antibody targeting an epitope on the hinge of the first and second amino-terminal insert (18F12). The latter was found to be conformation-dependent, suggesting structural differences between the Tau splicing isoforms and allowing insight in the roles played by the amino-terminal inserts. As this monoclonal antibody also has the capacity to detect tangle-like structures in different transgenic Tau mice and neurofibrillary tangles in brain sections of patients diagnosed with Alzheimer's disease, we also tested the diagnostic potential of 18F12 in a pilot study and found this monoclonal antibody to have the ability to discriminate Alzheimer's disease patients from control individuals based on increased Tau levels in the cerebrospinal fluid.

## Introduction

Tau is a neuronal microtubule-associated protein (MAP), primarily found in the axons, with major roles associated to assembly, stabilization and spacing of the microtubules, and thereby supporting axonal transport (Weingarten et al., 1975; Dixit et al., 2008). In the adult human brain, six Tau isoforms, with lengths ranging from 352 to 441 amino acids, can be expressed via alternative splicing of the microtubule-associated protein Tau (MAPT) transcript (Goedert et al., 1989). These isoforms are known to differ from each other by the presence of zero (0N), one (1N) or two inserts (2N) at the amino-terminus, and by the number of microtubule-binding repeat (MTBR) domains at the carboxy-terminal end, being either three (3R) or four (4R). Functionally, the MTBR are not only known to mediate the binding to the microtubules, but are also involved in the assembly of microtubules and the regulation of their dynamics (Feinstein and Wilson, 2005). While the function of the carboxy-terminal repeat regions is fairly well-known, the role of the amino-terminal part for Tau functioning remains poorly understood. Yet, this part, that extends outward from the microtubule surface, is involved in determining the spacing and stabilization of microtubules (Chen et al., 1992; Gustke et al., 1994; Feinstein et al., 2016) and allows the protein to interact with other signaling molecules and cellular constituents (Brandt et al., 1995; Liu et al., 2016).

Although Tau is considered to be a natively unfolded, heat-stable protein (Schweers et al., 1994), recent studies demonstrated that the protein can adopt to different conformations that can be modulated by post-translational modifications such as phosphorylation (Jeganathan et al., 2006, 2008; Mylonas et al., 2008; Mukrasch et al., 2009). Since phosphorylation also determines the propensity of Tau to assemble into filaments and neurofibrillary tangles, which is the main characteristic of a large group of neurodegenerative disorders known as Tauopathies (Buee et al., 2000; Sergeant et al., 2008), it is evident that conformational changes in Tau are closely linked to its pathobiology. Hence, insight in this link should provide the necessary guidelines for the development of tools allowing for accurate differential diagnosis between Tauopathies and effective therapeutic intervention (Fichou et al., 2019).

The assembly of Tau aggregates is the pathological hallmark for Tauopathies. However, these diseases vary in clinical and neuropathological representation and can be distinguished by which isoforms are incorporated into the insoluble aggregates. In AD, all six isoforms are integrated into the neuronal inclusions, while in progressive supranuclear palsy and corticobasal degenerations the inclusions primarily contain 4R Tau and in Pick's disease the Pick bodies consist only of 3R Tau (Buee and Delacourte, 1999). Different Tau inclusions also vary in size and morphology, leading to the hypothesis that Tauopathies are characterized by unique Tau strains formed by a specific combination of post-translational modifications on the Tau-splicing isoforms (Clavaguera et al., 2013; Guo and Lee, 2014; Boluda et al., 2015; Brettschneider et al., 2015; Goedert and Spillantini, 2017; Reilly et al., 2017). However, the underlying mechanism is still poorly understood. Gaining insight in this phenomenon will certainly be of major importance to unveil the structural basis for the Tau pathology.

In this study, we generated novel anti-Tau monoclonal antibodies (mAbs) using human Tau2N4R-ΔK280 isolated from humanized yeast as antigen. The use of *Saccharomyces cerevisiae* as Tau antigen producer has previously been proven successful (Rosseels et al., 2015), as the heterologous expressed human Tau undergoes pathologically relevant post-translational modifications, allowing the protein to undergo conformational changes and to self-assemble (Vandebroek et al., 2005, 2006; Vanhelmont et al., 2010). Our study yielded a phosphorylation-specific antibody (15A10), an antibody binding to the first MTBR (R1) (16B12), a carboxy-terminal antibody (20G10) and an antibody displaying higher affinity toward Tau2N isoforms in a seemingly conformation-dependent manner (18F12). Because 18F12 suggests structural differences among these Tau splicing isoforms, the latter was used in an explorative pilot study to test its capacity to detect Tau peptides in cerebrospinal fluid (CSF), thereby demonstrating its potency to discriminate Alzheimer's disease (AD) from non-AD patients.

## Methods

### Yeast Strains, Culture Conditions, and Tau Purification

The different BY4741 *S. cerevisiae* yeast strains used in this study for expression of Tau were obtained from the genome-wide yeast deletion collection. They were grown and selected on glucose-containing selective medium, according to standard procedures (Vandebroek et al., 2005). For each strain, the proper expression of Tau was confirmed by both Northern and Western blot analysis. For antigen production, we used the longest human Tau isoform (441 amino acids) containing an amino-terminal polyhistidine (His_6_) tag and the ΔK280 mutation (Tau2N4R-ΔK280), which is known to increase the aggregation propensity of Tau (Von Bergen et al., 2001). The protein was constitutively expressed in the yeast *pho85*Δ*adh1*Δ double deletion strain. The lack of the Pho85 kinase, which is the ortholog of human Cdk5, is known to trigger Tau hyperphosphorylation via the Gsk3β ortholog Mds1, as previously described (Vandebroek et al., 2005, 2006; Vanhelmont et al., 2010). The gene encoding the alcohol dehydrogenase Adh1 was deleted because we found this protein as a contaminant in the Tau preparations, which was problematic for mAb generation (Van Den Brande, 2014). The purification of human Tau2N4R-ΔK280 from the *pho85*Δ*adh1*Δ double deletion strain was performed with a nickel-immobilized metal affinity chromatography (Ni-IMAC) column (GE Health Care, Diegem, BE).

### Immunizations and Hybridoma Generation

Animal experiments were performed in compliance with European standards for the care and use of laboratory animals and experimental protocols were approved by the KU Leuven Animals Ethical Committee (project P055/2015, Leuven, BE).

Female SJL/J Rj mice were immunized by subcutaneously administering 10 μg human Tau2N4R-ΔK280 purified from *pho85*Δ*adh1*Δ yeast in complete Freund's adjuvant (Lonza, Verviers, BE). This was followed by a second intraperitoneal immunization with 10 μg purified Tau in incomplete Freund's adjuvant (Lonza, Verviers, BE). To generate high affine antibodies, three extra immunization and two booster sessions took place. Plasma was obtained through eye bleeding and anti-Tau titers were determined via an ELISA-based assay containing coated human Tau2N4R-ΔK280 (2 μg/ml). Plasma was added in a 1/10 dilution series, and detection was done using a rabbit anti-mouse antibody conjugated with horseradish peroxidase (HRP) (Nordic MUBio, Susteren, NL) followed by incubation with o-Phenylenediamine dihydrochloride (OPD) 99+% (Acros Organics, Geel, BE). A BioTek EL800 spectrophotometer (BioTek, Potton, UK) was used for plate reading at 490 nm. The spleen of the mouse exhibiting the highest anti-Tau titer was removed and washed in serum-free DMEM medium. A single cell suspension was obtained, after which cells were collected by centrifugation at 1,000 rpm for 10 min at room temperature. Spleen cells were fused with SP2/0 myeloma cells using polyethylene glycol, as described previously (Greenfield, 2018). Selection of the resulting hybridoma cells was done via the use of hypoxanthine-aminopterin-thymidine medium.

### Primary Antibody Screening

For the first selection of the generated hybridomas, we performed different ELISA assays using purified wild-type (WT) Tau2N4R purified from yeast or *Escherichia coli* as coated antigen (2 μg/ml). Detection was done as described above.

### Epitope Screening Assays

Epitope mapping of the novel mAbs was done using libraries of overlapping synthetic peptides (Pepscan, Lelystad, NL) (Langedijk et al., 2011). Two arrays were designed to map the epitopes. The first array consisted of 18 amino acid long non-phosphorylated peptides (Table S1) that covered the full sequence of human Tau2N4R and where each peptide has a 16 amino acids overlap with the former peptide. The second array contained phosphorylated peptides (Table S2) based on possible phosphosites as described in Sergeant et al. (2008). An example of the first 10 peptides of each peptide array are shown in Table 1. The binding capacity of the antibodies to the generated peptides was determined via a Pepscan-based ELISA. In short, an overnight incubation (4°C) with the primary antibody solution was followed by several washing cycles. Afterwards, the peptide arrays were incubated with a rabbit anti-mouse HRP conjugate (Southern Biotech, Uden, NL) for 1 h at 25°C and after several wash cycles, the peroxidase substrate 2,2′-azino-di-3-ethylbenzthiazoline sulfonate (ABTS) and H_2_O_2_ were added. After 1 h incubation, the colorimetric reaction was quantified.

**Table 1:** Different sets of peptides used for epitope mapping and phosphorylation-dependence studies.

**Phospho-independent**	**Sequence (first 10)**	**Phospho-dependent**	**Sequence (first 10)**
1	MAEPRQEFEVMEDHAGTY	1	pSPpSpSAKpSRLQpTAPVPMPD
2	EPRQEFEVMEDHAGTYGL	2	DEGAPGKQAAAQPHpTEIP
3	RQEFEVMEDHAGTYGLGD	3	APLVDEGAPGKQAAAQPH
4	EFEVMEDHAGTYGLGDRK	4	DLpSNVQpSKCGpSKDNIKHV
5	EVMEDHAGTYGLGDRKDQ	5	RENAKAKpTDHGAEIVpYKpS
6	MEDHAGTYGLGDRKDQGG	6	pSEKLDFKDRVQpSKIGpSLD
7	DHAGTYGLGDRKDQGGYT	7	GKVQIINKKLDLpSNVQpSK
8	AGTYGLGDRKDQGGYTMH	8	GDpTpSPRHLpSNVpSpSpTGpSID
9	TYGLGDRKDQGGYTMHQD	9	AAPPGQKGQANApTRIPAK
10	GLGDRKDQGGYTMHQDQE	10	LGNIHHKPGGGQVEVKpSE

To determine and reconfirm the established epitopes, competition ELISAs with wild-type or mutated peptides were designed (Table 2) (Pepscan, Lelystad, NL). Mutant peptides were generated by exchanging polar amino acids with the non-polar amino acid alanine to discriminate crucial amino acids necessary for binding. ELISA plates were coated with recombinant *E. coli*-produced human Tau2N4R and yeast-purified human Tau2N4R. 500 ng/ml of primary antibody was pre-incubated without or with either a wild-type or mutated peptide (50 μg/ml) for 2 h at room temperature and then added to the coated ELISA plate and an in-plate incubation step of 1 h at room temperature was performed. A rabbit anti-mouse IgG-HRP secondary antibody was added for detection for 1 h at room temperature. The colorimetric reaction using OPD was quantified by measuring the absorbance at 492 nm.

**Table 2:** Wild-type and mutated set of peptides used for competition ELISA design.

**16B12**	
PVPMPDLKNVKSKIGSTE	PVPMP**A**LKNVK**A**KIGSTE
**20G10**	
DSPQLATLADEVSASLAK	DSPQLA**A**LA**A**EVAASLAK
**18F12**	
TSDAKSTPTAEDVTAPLV	TSDAK**A**TP**A**AE**A**VTAPLV (Mutant peptide 1)
	TSDAK**A**TP**A**AEDVTAPLV (Mutant peptide 2)
	TSDAKSTP**A**AE**A**VTAPLV (Mutant peptide 3)
	TSDAKSTPTAE**A**VTAPLV (Mutant peptide 4)

### Immunoblotting on Total Yeast Extracts and Brain Extracts of Transgenic Mice and Humans

Total protein extracts from the wild type BY4741 strain and its isogenic *mds1*Δ and *pho85*Δ mutants expressing human Tau2N4R were processed by adding multiple protease inhibitors (cOmplete™ Mini, ethylenediaminetetraacetic acid (EDTA)-free Protease Inhibitor Cocktail (Sigma Aldrich, Overijse, BE), 250 mM Pefabloc® SC (Sigma Aldrich, Overijse, BE), 1 mg/ml Tosyl-L-lysyl-chloromethane hydrochloride (TLCK), 5 mM EDTA) and phosphatase inhibitors (100 mM NaF, 10 μM okadaic acid sodium salt (LC-laboratories, Woburn, MA, USA) and 0,2 mM Na_3_VO_4_). Cell lysis was accomplished using the FastPrep®-24 Classic Instrument (MP Biomedicals, Irvine, CA, USA). A complete set of the six Tau isoforms produced by *E. coli* was obtained from rPeptide (Watkinsville, GA, USA) and recombinant Tau2N4R was used as an internal reference.

Dephosphorylation studies of Tau were performed on purified Tau2N4R extracts from a *pho85*Δ*adh1*Δ yeast strain (Garcia-Sierra et al., 2003) using Lambda Protein Phosphatase according to the manufacturer (NEB, Ipswich, MA, USA).

Tris-buffered saline homogenates of the left cortex from non-transgenic mice (16 months old), transgenic APP-V717I mice (isoform 695 with London mutation; 16 months old; Moechars et al., 1999), transgenic Tau0N4R-P301S mice (5 months old; Allen et al., 2002) and transgenic Tau2N4R-P301L mice (9 months old; Terwel et al., 2005) were kindly provided by reMYND (Leuven, Belgium). A Tris-buffered sucrose extract of the hippocampus from a Tau1N4R-G272V/P301S mouse (12 months old; Schindowski et al., 2006) was kindly provided by the Lille University. Protein extracts from yeast and transgenic mice, used for SDS-PAGE and Western blot analysis, were made and processed as described previously, with a sodium dodecyl sulfate (SDS) sample buffer (50 mM Tris pH 8, 2% SDS, 0.1% bromophenol blue and 10% glycerol) containing β-mercaptoethanol as reducing agent (Vandebroek et al., 2005; Schindowski et al., 2006).

Human brain sample extracts were obtained from the Lille Neurobank at the University Hospital in Lille (frontal cortex, control cases and AD patients Braak stages 5/6). The sample collection was performed under reference DC-2008-642 with informed consent of the donors (CRB/CIC1403 Biobank, BB-0033-00030). Extracts were made in Tris-sucrose buffer while samples were processed in a lithium dodecyl sulfate sample buffer.

All antibodies used for detection of Tau are listed in Table 3. The generated antibodies in this study were diluted to a concentration of 1 μg/μl, unless specified otherwise. Immunodetection was performed using the Pico or Femto enhanced chemiluminescence (ECL) detection reagents from ThermoFisher Scientific (Waltham, MA, USA).

**Table 3:** Antibodies used in these studies.

**Antibody**	**Specificity**	**Source**
**Primary antibodies**		
ADx215	Tau; AA 16–24	ADx Neurosciences (Rosseels et al., 2015)
15A10	Tau; AA 197-207	Generated in this study
16B12	Tau; AA 249-255	Generated in this study
18F12	Tau; AA 67-73	Generated in this study
20G10	Tau; AA 423-433	Generated in this study
TAU5	Tau; aa 218–225	BD Pharmingen
BT2	Tau; aa 194-198	Thermofisher Scientific
Anti-1N Tau	Tau; STPTAEAEEAGI peptide	BioLegend
Anti-2N Tau	Tau; exon3	BioLegend
AT8	Tau; pSer^202^/pThr^205^	Thermofisher Scientific
ADx204	Tau; AA 6-18	ADx Neurosciences
**Secondary antibodies**		
Goat anti-mouse-HRP	Anti-mouse mAb	Biorad
Rabbit anti mouse-HRP	Anti-mouse polyclonal antibody	Southern Biotech
Biotinylated anti-mouse Rat	Anti-mouse antibody	Vector Laboratories

### Immunohistochemistry on Transgenic Mice and Human Brain Sections

For immunohistochemistry, we used free-floating coronal cryostat sections of the hippocampal and brainstem regions (40 μm) obtained from a wild-type, non-transgenic mouse and a Tau1N4R-G272V/P301S transgenic mouse (12 months old; Schindowski et al., 2006) and vibratome sections of the brainstem region (40 μm) obtained from a transgenic Tau0N4R-P301S mouse (Allen et al., 2002) and a transgenic Tau2N4R-P301L mouse (Terwel et al., 2005). Human biopsy samples (frontal cortex) consisted of 7 μm paraffin-embedded sections. Endogenous peroxidase activity was quenched by an H_2_O_2_ treatment for 30 min. Sections were either saturated by M.O.M.^TM^ Mouse IgG Blocking Reagent (Vector, Burlingame, CA, USA) or by normal horse serum (Vector, Burlingame, CA, USA). Overnight primary antibody incubation was done at 4°C and a biotinylated goat anti-mouse secondary antibody was used for detection. The signal was amplified using the VECTASTAIN ABC kit (Vector, Burlingame, CA, USA) and 3, 3′ diaminobenzidine tetrahydrochloride (DAB) revelation (Sigma Aldrich, Overijse, BE) was done in Tris 0.2M + H_2_O_2_.

The phosphorylation-specific antibody AT8 (pSer^202^/pThr^205^ Tau) (ThermoFisher Scientific, Waltham, MA, USA) served as reference antibody for immunohistochemistry.

### Development of a Novel Immunoassay and Pilot Study Using 18F12

The protocol of the novel immunoassay was based on Euroimmun's total Tau ELISA (Euroimmun, Lübeck, DE), where 18F12 and ADx204 were used as capture and detection antibodies, respectively. Recombinant human Tau2N4R purified from *E. coli* (rPeptide) was used as calibrator. Tau concentrations of the CSF samples were calculated using a four-parameter logistic curve fitting using the values assigned to the calibrator in order to convert the measured OD values to a concentration (pg/ml).

Cerebrospinal fluid (CSF) samples from 19 AD to 20 non-AD dementia patients were selected from the Biobank of the Institute Born-Bunge (Antwerp, BE), containing samples from patients recruited in the Hospital Network Antwerp (ZNA)-Middelheim and Hoge Beuken. This study was approved by the ethics committee of UAntwerp, Antwerp, BE (B300201420406). Informed consent was obtained from all subjects. All CSF samples were collected in polypropylene vials (Nalgene® catalog no. 5000-1020, ThermoFisher Scientific, Waltham, MA, USA), frozen in liquid nitrogen immediately after collection and stored at −80°C until analysis. AD and non-AD cases were selected based on a positive CSF biomarker profile being suggestive for AD or a negative biomarker profile being suggestive for non-AD (Dubois et al., 2014). A positive CSF biomarker profile was defined by a decreased amyloid beta 1-42 (Aβ1-42) concentration (<638.5 pg/mL) in combination with increased levels of total Tau (>296.5 pg/mL) and/or p-Tau181 (>56.5 pg/mL). These cut-offs had previously been determined in an autopsy-confirmed cohort (Engelborghs et al., 2008; Van Der Mussele et al., 2014). Statistical analysis was done by performing a non-parametric Mann-Whitney test.

## Results

### Generation and Characterization of the Novel mAbs

**Figure 1:**
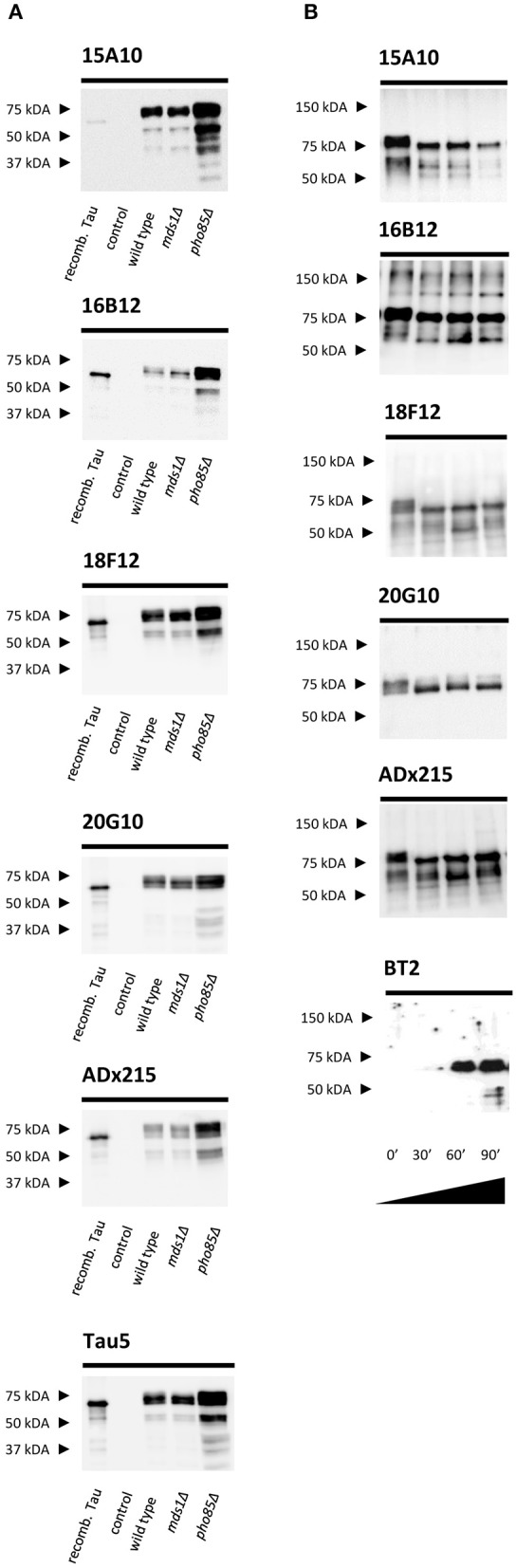
Characterization of the novel mAbs in yeast. **(A)** Western blot analysis of total protein extracts from different BY4741 yeast strains expressing human WT Tau2N4R using 15A10, 16B12, 20G10, 18F12, ADx215, and Tau5. *Lane 1*, bacterial recombinant protein Tau2N4R, *lane 2*, a BY4741 WT strain transformed with an empty vector (control), *lane 3*, a BY4741 WT strain expressing full-length human Tau2N4R, *lane 4*, a BY4741 *mds1*Δ strain expressing full-length human Tau2N4R, *lane 5*, a BY4741 *pho85*Δ expressing full-length human Tau2N4R. **(B)** Western blot analysis of the dephosphorylation study of LPP-treated BY4741 *pho85*Δ*adh1*Δ human Tau2N4R-ΔK280 samples using 15A10, 16B12, 20G10, 18F12, ADx215, and BT2. *Lane 1*, untreated extract of BY4741 *pho85*Δ*adh1*Δ strain expressing human Tau2N4R-ΔK280, *lanes 2-4*, BY4741 *pho85*Δ*adh1*Δ extracts treated with LPP for 30, 60, and 90 min, respectively.

We used purified human Tau2N4R-ΔK280 from the BY4741 *pho85*Δ*adh1*Δ yeast strain as antigen for mouse immunizations (Rosseels et al., 2015). For the selection of the generated hybridomas, we performed ELISA experiments using human Tau2N4R purified either from *E. coli* (no post-translational modifications) or yeast (post-translationally modified). These ELISA experiments led us to retrieve three mAbs (16B12, 18F12, and 20G10) that efficiently recognized recombinant human Tau2N4R from both *E. coli* and yeast and one mAb (15A10) that mainly reacted to yeast-purified human Tau2N4R. These data were then confirmed by SDS-PAGE and Western blot analysis using total protein extracts obtained from the yeast BY4741 wild-type strain and the *mds1*Δ or *pho85*Δ mutants, all of which were expressing human Tau2N4R, but displaying different Tau phosphorylation levels as previously reported (Vandebroek et al., 2005; Vanhelmont et al., 2010). Here, bacterial recombinant human Tau2N4R and a protein extract from a wild-type yeast strain expressing an empty vector served as controls. The four mAbs were capable of recognizing a banding pattern, ranging from 65 to 75 kDa, that represents different phospho-isoforms of monomeric Tau2N4R, as further confirmed by control Tau antibodies ADx215 and Tau5 (Rosseels et al., 2015) (Figure 1A). Consistent with the ELISA data, 15A10 showed only a very weak affinity for the bacterial recombinant Tau peptide, which suggested a certain phosphorylation-specificity of this mAb. The latter was tested by treating protein extracts from the Tau-expressing *pho85*Δ*adh1*Δ mutant with a protein phosphatase for different time intervals. As shown in Figure 1B, the capacity of 15A10 to detect Tau2N4R gradually decreased upon phosphatase treatment, while that of the control mAb BT2, which recognizes non-phosphorylated epitopes, increased. This indeed suggests that 15A10 binds to a phospho-epitope. In contrast, the overall signal intensities obtained with 16B12, 18F12, and 20G10 or the control mAb ADx215 were similar before and after phosphatase treatment, which indicates that their binding is independent of the phosphorylation status of Tau.

### Epitope Mapping

**Figure 2:**
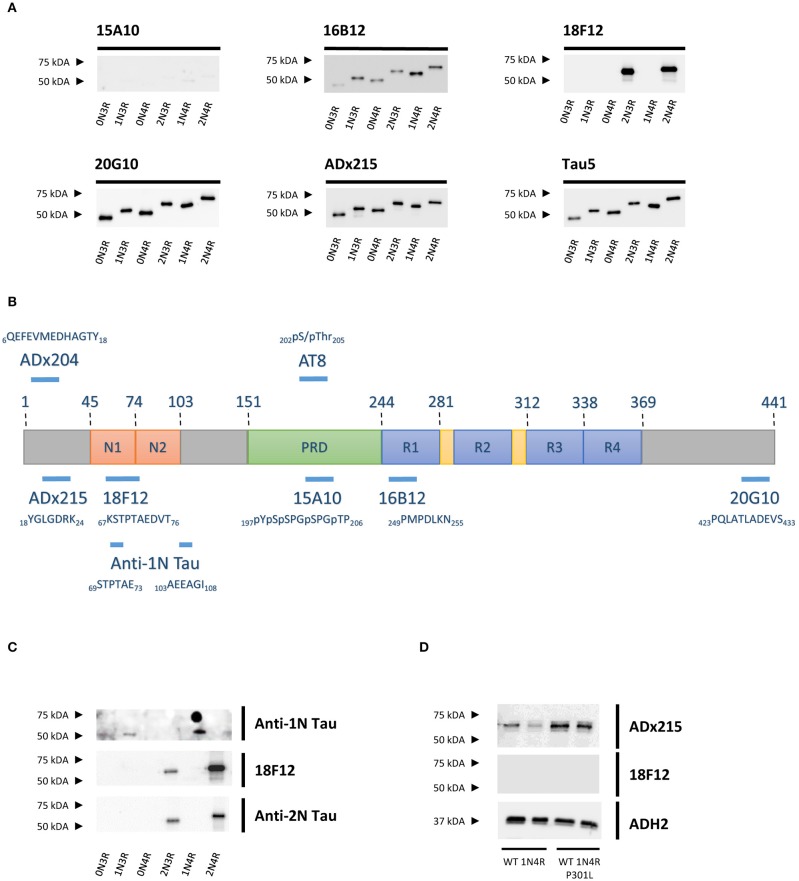
Characterization of the novel mAbs in *E. coli* and in yeast. **(A)** Western blot analysis of different bacterial recombinant proteins representing the six different splicing isoforms of Tau. *Lane 1*, human WT Tau0N3R, *lane 2*, human WT Tau1N3R, *lane 3*, human WT Tau0N4R, *lane 4*, human WT Tau2N3R, *lane 5*, human WT Tau1N4R, *lane 6*, human WT Tau2N4R. **(B)** Visual presentation of some of the used primary antibodies with their epitopes on full-length human Tau2N4R. **(C)** Western blot analysis of different bacterial recombinant proteins representing the six different splicing isoforms of Tau for comparison of 18F12 with an anti-1N Tau and an anti-2N Tau antibody. *Lane 1*, human WT Tau0N3R, *lane 2*, human WT Tau1N3R, *lane 3*, human WT Tau0N4R, *lane 4*, human WT Tau2N3R, *lane 5*, human WT Tau1N4R, *lane 6*, human WT Tau2N4R. **(D)** Western blot analysis of total protein extracts from WT BY4741 yeast strain expressing either WT human Tau1N4R or Tau1N4R-P301L using 18F12, ADx215, and ADH2 as a control. *Lane 1–2*, human WT Tau1N4R, *lane 3–4*, human Tau1N4R-P301L.

**Figure 3:**
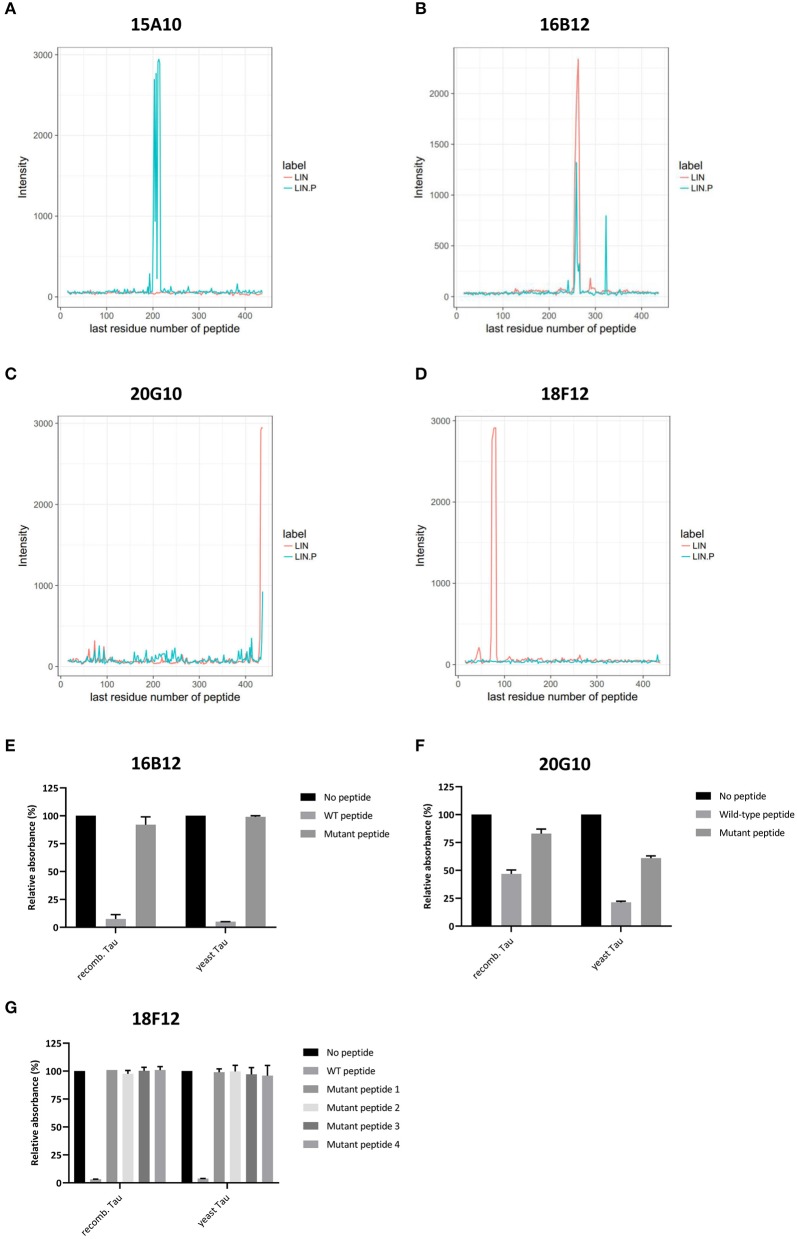
Delineation of epitopes using an ELISA-based peptide array scanning method performed by Pepscan. **(A–D)** Linear intensity profile recorded for **(A)** 15A10, **(B)** 16B12, **(C)** 20G10, and **(D)** 18F12. The overlay of intensity profiles recorded on the non-phosphorylated peptide set (red) and the phosphorylated peptide set (blue) is plotted in the same graph to allow direct comparison between responses of the antibody to the different peptide arrays. **(E–G)** Competition ELISAs for **(A)** 16B12, **(B)** 20G10, and **(C)** 18F12 with their respective wild-type and mutated epitope-containing peptides. Amino acid sequences of the used peptides are listed in Table 2. Data are shown as the relative absorbance, with the signal of the antibody without epitope-containing peptide being the reference value. Reactivity of the antibodies was tested toward recombinant E. coli Tau2N4R (recomb. Tau) and yeast-purified human Tau2N4R (yeast Tau).

To identify the epitopes of our novel mAbs in more detail, we first evaluated the reactivity of the mAbs toward the six different Tau splicing isoforms as produced in *E. coli* (Figure 2A). We used the pan-Tau mAb Tau5 as control. As expected, 15A10 weakly detected any of the recombinant isoforms, which again confirmed its phosphorylation-dependency. Two other mAbs, i.e., 16B12 and 20G10, detected all six isoforms and although they displayed different affinities, it was clear that these mAbs recognize epitopes that are shared amongst the different splicing isoforms. The 18F12 mAb detected only Tau2N3R and Tau2N4R, suggesting that its epitope would be present in the second amino-terminal insert. To confirm this and to further delineate each epitope, an ELISA-based peptide array scanning was performed at Pepscan (Langedijk et al., 2011). We used two arrays consisting of either partially overlapping non-phosphorylated peptides (Table S1) or phosphorylated peptides (Table S2), thereby covering the entire Tau2N4R sequence. 15A10 was found to bind a sequence located in the proline-rich domain but only when this is phosphorylated, i.e., _197_pYpSpSPGpTP_207_ (Figure 3A), thereby settling its phosphorylation-dependency (Figure 2B). 16B12 and 20G10 both recognized linear unphosphorylated peptides, respectively _249_PMPDLKN_255_, which is found in the first MTBR R1, and _423_PQLATLADEVS_433_, which is located in the carboxy-terminus of the protein (Figures 3B,C). Similar as for 15A10, their epitopes are commonly present in the different splicing isoforms of Tau. Most interesting, the epitope for 18F12 mapped to the unphosphorylated peptide _67_KSTPTAEDVT_76_ (Figure 3D), which corresponds to the last seven residues of the first amino-terminal insert and the first three residues of the second amino-terminal insert. The latter seems to define the specificity of the antibody because our Western blot analysis indicated that this antibody failed to detect recombinant Tau1N3R or Tau1N4R even though the majority of the epitope is found in the first amino-terminal insert. One possible explanation is that the second amino-terminal insert induces a specific conformational change that is required to unmask the 18F12 epitope. If true, this means that the Tau2N3R and Tau2N4R splicing isoforms are characterized by a specific intramolecular fold. To further confirm this, we performed an additional Western blot analysis using a commercial anti-1N Tau antibody (BioLegend, San Diego, CA, USA) of which the epitope overlaps partially with 18F12, but corresponding in sequence to the border of the first amino-terminal insert as present in the Tau1N3R and Tau1N4R isoforms. As control we also used a commercial anti-2N Tau antibody that detects the sequence encoded by exon3 (BioLegend, San Diego, CA, USA). The anti-1N Tau antibody clearly solely visualized the recombinant Tau1N3R and Tau1N4R isoforms, while 18F12 and the anti-2N Tau antibody detected solely the Tau2N3R and Tau2N4R isoforms (Figure 2C).

Previously, we reported that the P301L mutation has an impact on Tau folding as judged by the observation that it significantly reduces the immunoreactivity of the mAb PG5, an antibody that recognizes P-S409, which is an epitope separated by 108 amino acids from the site of the mutation (Vanhelmont et al., 2010). Therefore, we introduced the P301L mutation into the Tau1N4R isoform to elucidate whether this mutation would also influence the presumed folding and the exposure of the 18F12 epitope. As shown in Figure 2D, we indeed observed that for mAb ADx215 signal intensities were increased in the Tau1N4R-P301L mutant, pointing to better accessibility of the antibody to its epitope on the Tau protein. However, such improvement was not observed when using 18F12.

To reconfirm the former established epitopes of 16B12, 20G10, and 18F12, competition ELISAs were designed. Plates were coated with recombinant *E. coli*-produced human Tau2N4R or yeast-purified human Tau2N4R and the novel mAbs were pre-incubated without or with a wild-type or mutated peptide. These peptides covered the minimal epitopes, as defined from the previous peptide arrays, and some adjacent amino acid residues. The mutations comprised the exchange of polar amino acids with the non-polar amino acid alanine (Table 2). When mAb 16B12 was pre-incubated with the wild-type peptide, signal intensities were absent, pointing to full binding of the mAb to the generated peptide. In contrast, when pre-incubated with the mutant peptide, signal intensities were comparable to those obtained in the absence of a competing peptide, thus allowing full binding of 16B12 to the coated human Tau2N4R (Figure 3E). This means that the exchange of the polar amino acids for alanine in the mutant peptide caused full disruption of the epitope of 16B12. When 20G10 was pre-incubated with the wild-type peptide, the signal intensity dropped, but no full signal loss was observed. Pre-incubation with the mutant peptide did not yield full signal recovery (Figure 3F). This indicates that 20G10 displays a higher affinity for the full-length Tau2N4R protein than for the competing peptides, that further optimization of the ELISA protocol is required to clarify whether this is due to contextual differences between the full-length protein and the competing peptides, for instance that the 20G10 epitope is not completely covered by the latter, and that surrounding sequences are required for its optimal binding. For 18F12, we used four different mutant peptides in order to determine in which amino-terminal insert the crucial amino acids for its binding are found. However, while a complete signal loss was seen upon pre-incubation with the wild-type peptide, each of the mutant peptides failed to prevent the binding of 18F12 to the coated human Tau2N4R (Figure 3G). This indicates that the sequences encoded by exon2 and exon3 are equally important to constitute the 18F12 epitope.

### Biochemical Validation of the New mAbs in Transgenic Mouse Models and Human Brain

**Figure 4:**
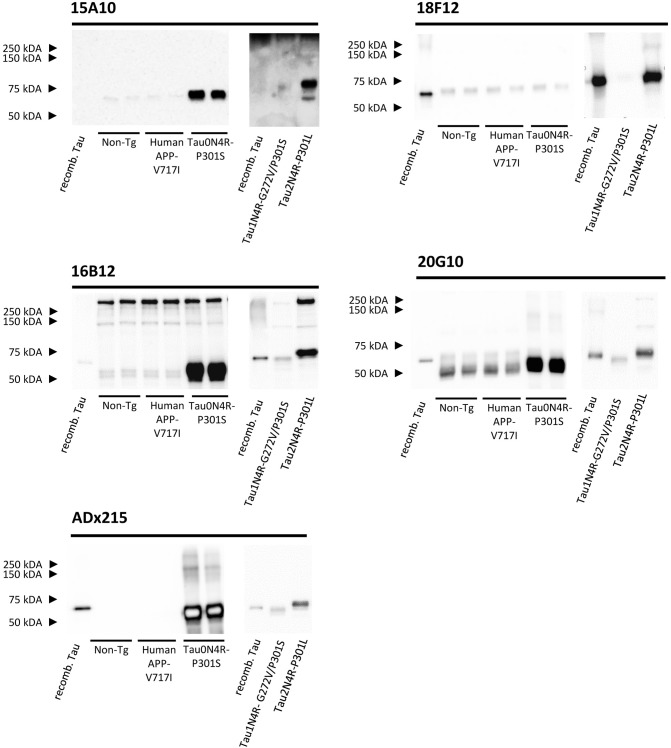
Biochemical validation of the novel mAbs in transgenic mouse brain extracts. Western blot analysis of left cortex brain extracts obtained from non-transgenic mice (*Non-Tg*), transgenic human APP-V717I mice, transgenic human Tau0N4R-P301S, Tau1N4R-G272V/P301S, and Tau2N4R-P301L mice using 15A10, 16B12, 18F12, 20G10, and ADx215. *Lane 1*+*8*, bacterial recombinant protein human WT Tau2N4R, *lane 2–3*, non-transgenic mouse brain extracts, *lane 4–5* transgenic human APP-V717I mouse brain extracts, *lane 6–7*, transgenic human Tau0N4R-P301S mouse brain extracts, *lane 9*, transgenic 1N4R-G272V/P301S mouse brain extracts, *lane 10*, transgenic human Tau2N4R-P301L mouse brain extracts.

None of the epitopes defined above are human-specific since they are also present in Tau isoforms of rodents. These findings were confirmed by immunoblot experiments performed on cortex homogenates of non-transgenic and transgenic mice expressing human APP-V717I. Indeed, all mAbs recognized a 55 kDa endogenous murine Tau isoform, albeit with different low affinities, while the control mAb ADx215, which is a human-specific Tau mAb (Rosseels et al., 2015), did not (Figure 4). In addition to this, 16B12 was also found to cross-react with other murine proteins of higher molecular weight (approximately between 150 and 250 kDa).

As our results with *E. coli* recombinant Tau indicated variable reactivity of the mAbs toward the different human Tau splicing isoforms, we additionally tested the mAbs on cortex homogenates of transgenic Tau0N4R-P301S, Tau1N4R-G272V/P301S, or Tau2N4R-P301L mice. Notably, all Tau transgenic mice contained a P301 mutation. Since no information is available that allows comparing the expression levels of the human transgene in these mice, we used the human-specific mAb ADx215 as a reference in this analysis. Based on this, it was clear that 15A10, 16B12, and 20G10 detected all three transgenes with affinities comparable to those of ADx215 (Figure 4). The exception was again 18F12, as this mAb easily detected human Tau2N4R-P301L but failed to stain Tau0N4R-P301S and Tau1N4R-G272V/P301S, even when blots were overexposed as shown for Tau1N4R-G272V/P301S in Figure 4. As such, these data correspond to those obtained with the recombinant proteins purified from *E. coli*. and they again suggest that the structural properties of the Tau protein vary between the different splicing isoforms.

**Figure 5:**
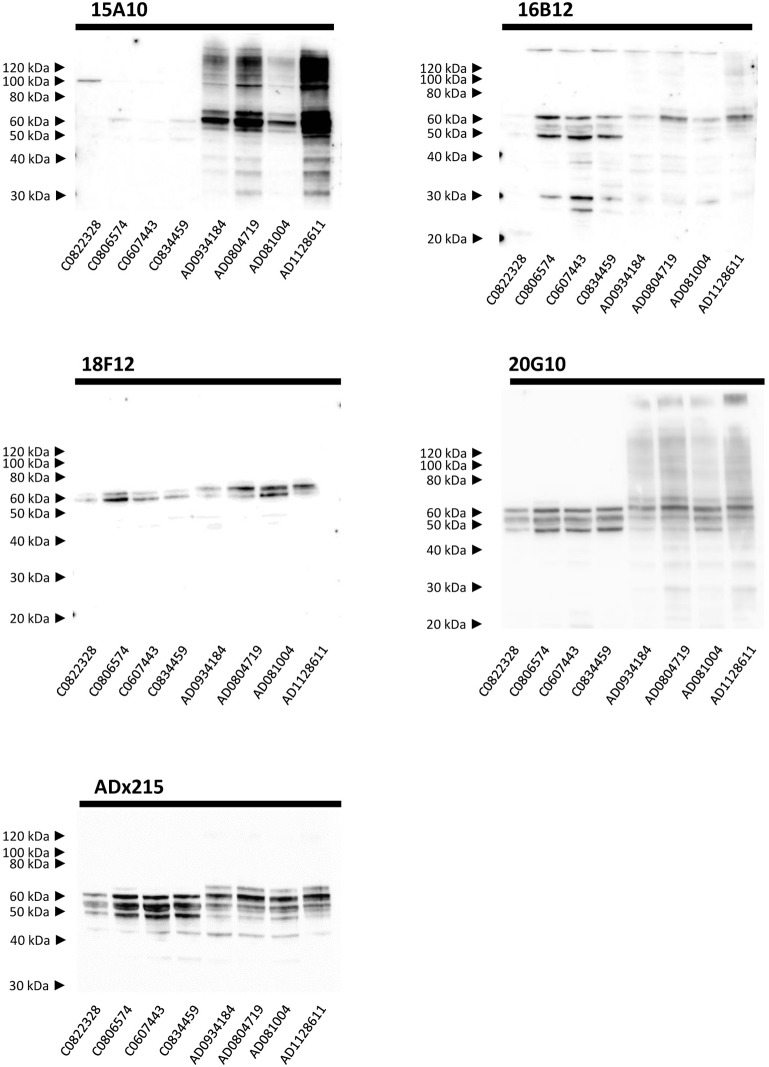
Biochemical validation in human brain extracts. Western blot analysis of homogenates of human frontal cortex. *Lane 1–4*, brain extracts from healthy controls (C numbers), *lane 5–8* brain extracts from AD patients (AD numbers) at Braak stage 5.

Next, we performed Western blot analysis on human frontal cortex homogenates, obtained from post-mortem biopsies of healthy controls and AD patients at Braak stage 5/6. As shown, each mAb yielded a different pattern (Figure 5). 15A10 only weakly detected monomeric Tau in the control samples but it clearly labeled the previously reported hyperphosphorylated Tau triplet (Sergeant et al., 2008) in the different AD samples. In addition, 15A10 also detected higher-order oligomeric Tau species in all the AD cases. 16B12 was able to detect different monomeric Tau isoforms in the control samples but mainly visualized the 64 kDa band and faintly the 69 kDa band of the triplet in AD samples. 18F12 labeled a doublet in both the control and AD samples but in the latter, the doublet ran at slightly higher molecular weight, which is likely due to Tau hyperphosphorylation. Intriguingly, the stained doublet corresponds to the 60 kDa and 64 kDa bands of the triplet, which in size matches best with the phosphorylated Tau1N3R and Tau2N3R, or Tau1N4R peptides, respectively. The largest Tau2N4R isoform was not detected, probably because its expression in normal or diseased adult brain is known to be very low (Goedert and Jakes, 1990; Buee et al., 2000). Finally, 20G10 gave similar staining as that of the control mAb ADx215 with a labeling of different monomeric Tau isoforms in the controls and the typical Tau triplet in addition to a 48 kDa band in AD patients.

### Immunohistochemical Validation in Transgenic Mouse and Human AD Brain

In addition to Western blot analysis, we also tested the performance of the mAbs in immunohistochemical stainings on brain sections of transgenic mice as well as sections obtained from AD patients and healthy controls. In both cases, the mAb AT8 served as reference.

**Figure 6:**
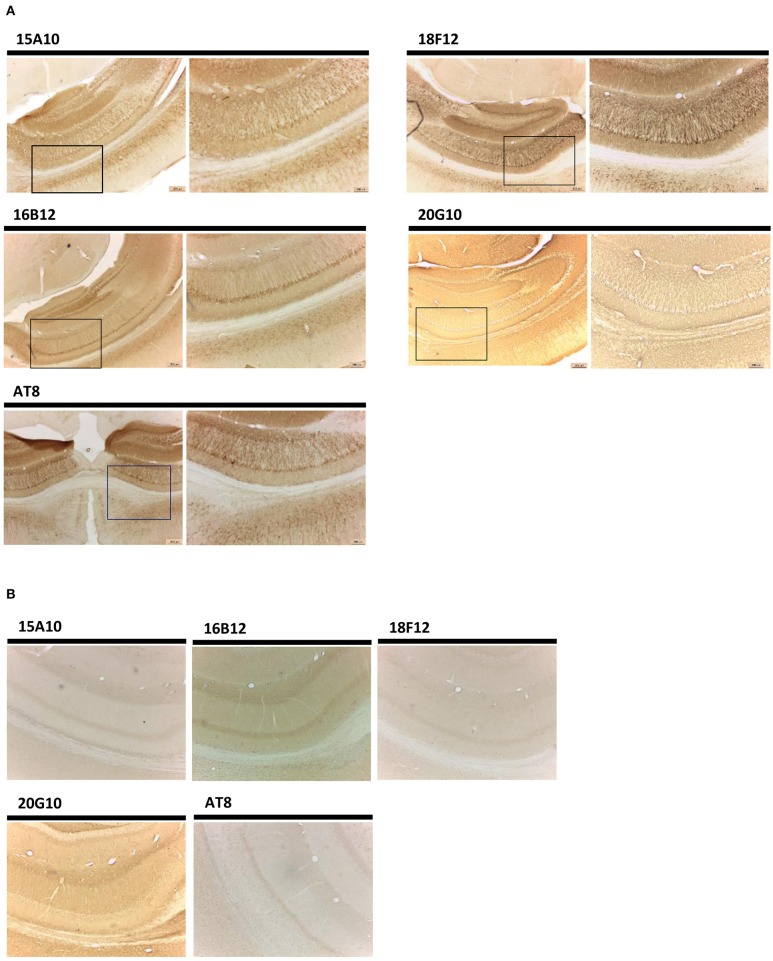
Immunohistochemistry in transgenic mouse hippocampal brain sections. **(A)** Pictures represent immunohistochemical stainings performed using mAbs 15A10, 16B12, 18F12, and 20G10 on different regions in the hippocampus of Tau1N4R-G272V/P301S mouse brain. AT8 was used as a reference antibody. Lower magnification pictures are shown on the left, while the corresponding higher resolution images are shown on the right. **(B)** Pictures represent immunohistochemical stainings performed using mAbs 15A10, 16B12, 18F12, and 20G10 on different regions in the hippocampus of wild-type non-transgenic mouse brain. AT8 was used as a reference antibody.

We first tested our mAbs on sections of the hippocampal region obtained from a 12 months old transgenic Tau1N4R-G272V/P301S mice (Schindowski et al., 2006). All four generated mAbs were able to detect filamentous Tau and tangle-like Tau pathology to various extents (Figure 6A). For 18F12, this was rather surprising as the human transgene was a Tau1N4R species, which according to the data described above is missing the 18F12 epitope. For 20G10, we observed a high background staining in comparison to the other mAbs, which is probably due to its higher affinity toward endogenous murine Tau as indicated by our Western blot analysis. When the antibodies were tested on a brain section of a wild-type, non-transgenic mouse, no tangle-like pathology was detected (Figure 6B).

**Figure 7:**
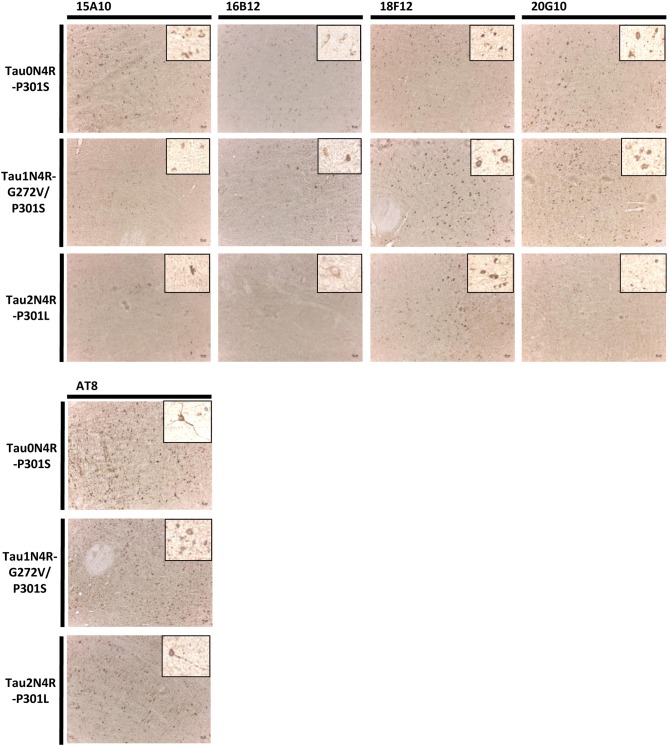
Immunohistochemistry in transgenic mouse brain stem sections. Pictures represent immunohistochemical stainings performed using mAbs 15A10, 16B12, 18F12, and 20G10 on the brain stem of Tau0N4R-P301S, Tau1N4R-G272V/P301S, and Tau2N4R-P301L transgenic mice. AT8 was used as a reference antibody. A magnification of the tangle-like Tau pathology is also shown.

In the next experiment, we performed immunohistochemical analysis on the brainstem region, but this time also included the Tau0N4R-P301S mouse (Allen et al., 2002) and the transgenic Tau2N4R-P301L mouse (Terwel et al., 2005). Again, each of the mAbs detected Tau pathology in the different transgenic mice, even 18F12 for which its epitope is completely absent in the Tau0N4R-P301S transgene (Allen et al., 2002) (Figure 7). However, it should be noted that the 18F12 epitope is present in murine Tau (see Figure 4) and thus perhaps the data can be explained if a fraction of murine Tau would become trapped during the oligomerization process of the human transgene.

**Figure 8:**
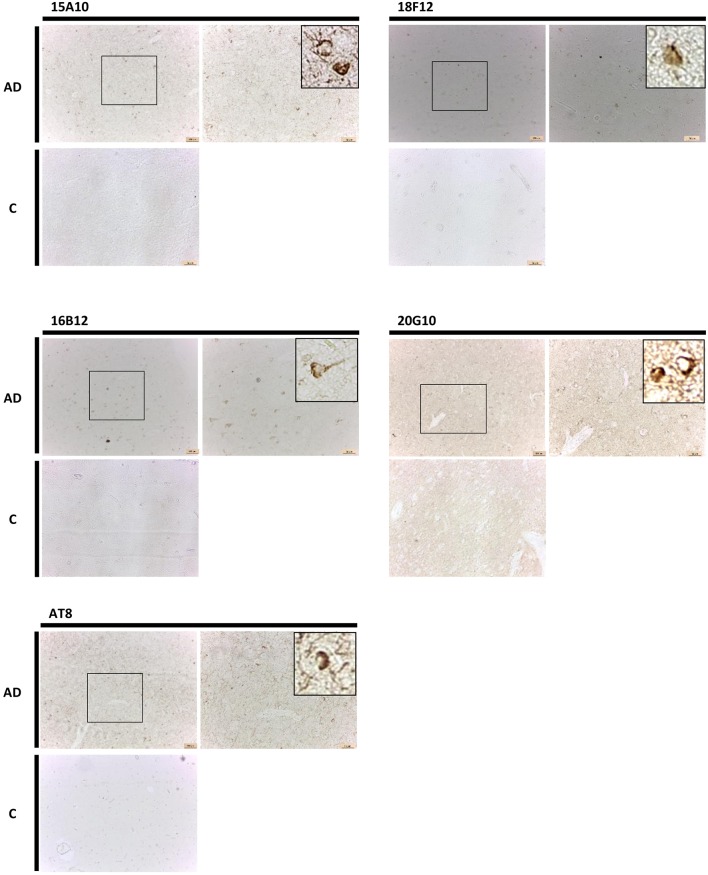
Immunohistochemistry in human brain sections. Pictures represent immunohistochemical stainings performed using mAbs 15A10, 16B12, 18F12, and 20G10 on different regions of the cortex of human control subjects (C) and AD patients. Also shown is a magnification of the tangles detected.

Finally, also in human brain, all mAbs were able to visualize pathological pre-tangles and neurofibrillary tangles in the cortex of AD-patients, though 15A10, 16B12, and 18F12 were more efficient than 20G10 (Figure 8).

### Diagnostic Potential of Novel Immuno-Assay Making Use of 18F12

**Figure 9:**
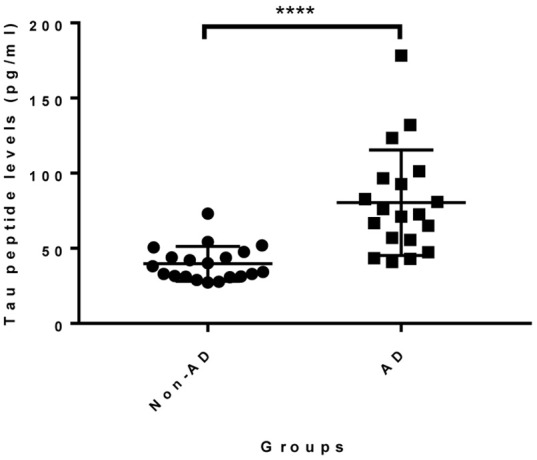
Diagnostic potential of novel immuno-assay in CSF. Scatter dot plot of levels of a pilot study measuring Tau peptide levels in CSF obtained from non-AD (*n* = 20) and AD patients (*n* = 19). The *line* presented in each dot plot is the median level, while *bars* show the interquartile range. ****p <0.0001.

Given that the epitope of 18F12 appears to be conformation-dependent and that the mAb has the ability to detect tangle-like structures in transgenic mice and neurofibrillary tangles in human brain, we tested the diagnostic potential of 18F12 in an explorative pilot study using CSF samples of 19 AD patients and 20 non-AD patients. To this end, we developed a Tau ELISA assay that included recombinant *E. coli* Tau 2N4R as calibrator and used 18F12 as capture antibody and biotin-labeled ADx204 as detection antibody in order to specifically quantify Tau peptides. As depicted in Figure 9, the detected Tau peptide levels were significantly increased in the CSF samples of AD patients in comparison to those found in samples of non-AD patients (*p* <0.0001). S/N values ranged between minimally 3,7 and maximally 22,2 (Table 4).

**Table 4:** S/N values novel immuno-assay 18F12.

**Patient**	**S/N**	**Patient**	**S/N**
HB04110	7,4	HB04387	5,5
HB04190	6,2	HB04252	17,0
HB04127	9,9	HB04311	7,1
HB04723	7,1	HB04395	16,0
HB04689	4,7	HB04279	4,9
HB04045	8,2	HB04244	5,3
HB04561	6,6	HB04301	5,3
HB04552	9,4	HB04532	6,4
HB04636	8,0	HB04326	22,2
HB04173	5,2	HB04775	12,9
HB04204	13,4	HB04465	5,1
HB04529	11,0	HB04215	6,7
HB04398	7,6	HB04413	5,2
HB04399	6,3	HB04560	6,3
HB04579	6,5	HB04141	10,0
HB04237	5,1	HB04044	8,4
HB04359	9,2	HB04438	8,1
HB04114	10,0	HB04150	9,0
HB04475	5,1	HB04550	3,7
HB04414	6,0		

## Discussion

Previously, we demonstrated that human Tau is phosphorylated at the same epitopes as found in AD brain when expressed in the yeast *S. cerevisiae* and that, also in this model, hyperphosphorylation triggers conformational changes in Tau thereby leading to a small fraction of sarkosyl insoluble material (Vandebroek et al., 2005, 2006; Vanhelmont et al., 2010). We also showed earlier that the yeast model provided a good source of post-translationally modified antigen for the production of novel high-affinity mAbs (Rosseels et al., 2015). For the current study, we purified human Tau2N4R-ΔK280 from a *pho85*Δ*adh1*Δ yeast strain and used this antigen to generate several additional mAbs, four of which were characterized in more detail. Our studies demonstrated this set of mAbs to include a phosphorylation-dependent antibody (15A10), an antibody binding to the first MTBR (16B12), a very sensitive carboxy-terminal antibody (20G10) and, most interestingly, an antibody that showed higher affinity toward Tau2N isoforms in comparison to Tau1N isoforms, even though most of its epitope is located on exon2 (18F12). The latter result may suggest that the affinity of 18F12 is based on a conformational difference between Tau1N and Tau2N isoforms, making the epitope more accessible whenever the second amino-terminal insert is included. We believe that the latter is an interesting asset, not only because we demonstrated the capacity of 18F12 to discriminate AD patients from non-AD individuals based on CSF ELISA, but also because this mAb allows insight in the structural differences between Tau splicing isoforms and the roles played by the amino-terminal inserts.

Indeed, ever since it became clear that Tau was not a natively unfolded protein as initially thought (Schweers et al., 1994), but that it contained sequence motifs in the MTBR domain with an enriched propensity to adopt a β-sheet structure that promote the self-assembly of Tau (Von Bergen et al., 2000; Mukrasch et al., 2005), studies on structure and function have mainly focused on the carboxy-terminal half of the protein. Moreover, this focus is also explained by the fact that the presence or absence of the fourth MTBR is disease-specific (Buee et al., 2000; Sergeant et al., 2008) and that it determines the structural differences typifying the filaments seen in different Tauopathies (Fitzpatrick et al., 2017; Falcon et al., 2019; Goedert et al., 2019). Nonetheless, the amino-terminal half of protein Tau, also known as the projection domain, is associated to some interesting features. Indeed, it plays a role in the formation and organization of the microtubule network, thereby acting in concert with the MTBR domain (Chen et al., 1992; Gustke et al., 1994; Feinstein et al., 2016). In addition, it allows Tau to interact with other signaling proteins (Liu et al., 2016) and it contains a phosphatase-activating domain at the N-terminus that controls the anterograde fast axonal transport and is aberrantly exposed in disease-related forms of Tau (Kanaan et al., 2011; Combs et al., 2016). The projection domain also determines the axodendritic sorting of Tau (Zempel et al., 2017) and it allows for interaction of Tau with the axonal plasma membrane (Brandt et al., 1995), a feature modulated by splicing regulation of exon2 and exon3 (Arikan et al., 2002; Li et al., 2003). The presence of the first amino-terminal insert encoded by exon2 inhibits Tau secretion (Kim et al., 2010) and, consistently, targeting the amino-terminus, or the amino-terminal insert region, with antibodies prevents Tau seeding and spreading as shown in cell-based assays (Apetri et al., 2018) and in passive immunization studies on transgenic mice (Dai et al., 2015, 2018). Notably, one of the antibodies used in these studies, i.e., CBTAU-28.1 (Apetri et al., 2018) recognizes an epitope that overlaps with the one of 18F12, making it likely that the latter also has the capacity to inhibit the propagation of the Tau pathology.

However, even though amino-terminal directed antibodies seem to have this capacity, there are also *in vitro* and *in vivo* studies describing an antibody directed to central Tau, “antibody D” (AA 235-250), that showed greater efficiency in inhibiting Tau seeding and spreading when compared to “antibody A”, which is directed to the N-terminus of Tau (AA 15-24) (Courade et al., 2018; Albert et al., 2019). These data thus stress the necessity of the selection of the Tau epitope, which will be a crucial factor when therapeutic efficacy needs to be determined. Our novel generated mAb 16B12 has 2 AA in common with the epitope of antibody D, which could be an incentive to also test its therapeutic potential.

Recent NMR, FRET, and small-angle X-ray scattering analyses revealed that protein Tau acquires different conformations based on intermolecular interactions between different regions, including the amino-terminal sequences, which are altered by post-translational modification (Jeganathan et al., 2006, 2008; Mylonas et al., 2008; Mukrasch et al., 2009). These studies clarify some of our observations on the performance 18F12. When performing Western blot analysis we observed that 18F12 fails to detect Tau1N peptides but readily detects Tau2N peptides in protein extracts obtained from bacteria, yeast, and mice. This suggests that when Tau is expressed in these hosts, the 18F12 epitope appears to be masked in the absence of the second amino-terminal insert but exposed in its presence. The NMR analysis demonstrated that the first amino-terminal insert can interact with a sequence motif between residues S113 and Q124 as well as with the residue stretches in proline-rich domains spanning the region between I151 and Q244 (Mukrasch et al., 2009). In addition, that study also showed that the second amino-terminal insert brings an additional β sheet formed by residues G86 until Q92 and a small hydrophobic stretch exactly at its boundary with the first amino-terminal insert, which corresponds to a region where both inserts tend to fold back onto each other (Mukrasch et al., 2009). Hence, by adding distance restraints for interactions in which the first amino-terminal insert is involved, and by altering the local conformation at the junction of the two amino-terminal inserts, the insertion of the second amino-terminal insert may indeed enhance exposure of the 18F12 epitope. Another observation we made is that in contrast to bacteria, yeast or mice, 18F12 readily recognized the Tau1N peptides in immunoblots of extracts obtained from human biopsies as well as in immunohistochemical detection of tangles in brain sections of transgenic mice and human AD patients. This suggests that the exposure of the 18F12 epitope is dependent on an intermolecular interaction or a post-translational modification that is present in the post-mortem human biopsies and in transgenic mice and is only occurring when Tau starts to form filaments and tangles. Notably, the first amino-terminal insert contains seven phosphorylation sites, one of which is located in the 18F12 epitope, i.e., residue T69, and is a known target of the MAP kinase ERK2 as well as Gsk3β (Godemann et al., 1999; Sergeant et al., 2008; Qi et al., 2016). Finally, we also showed in this study that, at least in yeast, the introduction of the P301L mutation in the Tau1N4R isoform enhanced the accessibility of the ADx215 epitope at the very amino-terminal end. In contrast, this was not the case for the epitope of 18F12. This suggests that the P301L mutation, which is located outside of the proline-rich domain, does affect the overall folding of Tau but without having an effect on the accessibility of the 18F12 epitope.

Besides the high variety of conformations adopted by monomeric Tau, there is also the diversity of pathological representation among the different Tauopathies. This diversity is thought to be attributed to unique Tau strains formed by the cross-talk between post-translational modifications and leading to the distinct Tau conformations that specify the structural differences of the deposited Tau filaments (Clavaguera et al., 2013; Guo and Lee, 2014; Boluda et al., 2015; Brettschneider et al., 2015; Goedert and Spillantini, 2017; Reilly et al., 2017). This heterogeneity of the deposits is evidenced by their different sensitivity when treated with proteases (Taniguchi-Watanabe et al., 2016). As such, conformational-dependent antibodies can help to identify the unique arrangements of Tau filaments among Tauopathies.

18F12 is not the first conformation-dependent antibody raised against Tau, but it completes a list that includes MC1, Alz50, Tau-66, MN423, SMI34, TOC-1, TOMA, GT-7, and GT-38 (Carmel et al., 1996; Jicha et al., 1997a,b; Ghoshal et al., 2001; Garcia-Sierra et al., 2003; Skrabana et al., 2004; Castillo-Carranza et al., 2014; Ward et al., 2014; Gibbons et al., 2018, 2019), and all these antibodies helped to get insight in the relation between the structure and pathobiology of protein Tau. Moreover, the antibodies GT-7 and GT-38 have recently been shown to selectively bind Tau in AD (Gibbons et al., 2018, 2019). Whether this would also be the case for 18F12 remains to be investigated, but it is interesting to note that a previous study demonstrated the presence of the first amino-terminal insert in neurofibrillary structures already at early Braak stages of AD (Soltys et al., 2005), which suggests that 18F12 might be a good asset for early diagnosis of Tauopathy.

## Data Availability Statement

All datasets generated for this study are included in the article/Supplementary Material.

## Ethics Statement

The studies involving human participants were reviewed and approved by (1) Reference DC-2008-642 - (CRB/CIC1403 Biobank, BB-0033-00030) (2) Ethics committee of UAntwerp, Antwerp, BE (B300201420406). The patients/participants provided their written informed consent to participate in this study. The animal study was reviewed and approved by KU Leuven Animals Ethical Committee (project P055/2015, Leuven, BE).

## Author Contributions

JW and DT supervised the project. JV, SEd, DV, EDS, JR, SM, and CF performed experiments and analyzed data. MB, SEn, ML, SC, and LB kindly provided study material. JV, JW, and DT prepared the manuscript. NG, VF, ES, MB, SEn, LB, and EV revised the manuscript.

### Conflict of Interest

The authors declare that the research was conducted in the absence of any commercial or financial relationships that could be construed as a potential conflict of interest.
