# Supplementary material for: A Novel Tau Antibody Detecting the First Amino-Terminal Insert Reveals Conformational Differences Among Tau Isoforms
Source: Front Mol Biosci. 2020 Mar 31;7:48. doi: 10.3389/fmolb.2020.00048 (PMC7136581; doi:10.3389/fmolb.2020.00048)
Supplement: Supplementary file 1 [file Table_1.docx]

**Supplementary Data**

**Table S1. Linear peptide sequences used for epitope mapping and raw data of signal intensities for each of the four monoclonal antibodies towards linear peptides covering the amino acid sequence of human Tau2N4R.** Each linear peptide was 18 amino acids long with an overlap of 16 amino acids with the former linear peptide.

| **Linear peptide sequence** | **15A10** | **18F12** | **20G10** | **16B12** |
| --- | --- | --- | --- | --- |
| MAEPRQEFEVMEDHAGTY | 56 | 56 | 65 | 37 |
| EPRQEFEVMEDHAGTYGL | 54 | 23 | 79 | 40 |
| RQEFEVMEDHAGTYGLGD | 47 | 12 | 61 | 29 |
| EFEVMEDHAGTYGLGDRK | 55 | 29 | 62 | 43 |
| EVMEDHAGTYGLGDRKDQ | 35 | 25 | 58 | 32 |
| MEDHAGTYGLGDRKDQGG | 53 | 37 | 77 | 40 |
| DHAGTYGLGDRKDQGGYT | 58 | 39 | 100 | 43 |
| AGTYGLGDRKDQGGYTMH | 44 | 16 | 56 | 32 |
| TYGLGDRKDQGGYTMHQD | 50 | 26 | 66 | 20 |
| GLGDRKDQGGYTMHQDQE | 35 | 31 | 101 | 31 |
| GDRKDQGGYTMHQDQEGD | 49 | 53 | 95 | 38 |
| RKDQGGYTMHQDQEGDTD | 54 | 61 | 91 | 39 |
| DQGGYTMHQDQEGDTDAG | 51 | 91 | 54 | 28 |
| GGYTMHQDQEGDTDAGLK | 42 | 133 | 60 | 40 |
| YTMHQDQEGDTDAGLKES | 46 | 176 | 62 | 39 |
| MHQDQEGDTDAGLKESPL | 48 | 211 | 32 | 24 |
| QDQEGDTDAGLKESPLQT | 47 | 162 | 35 | 22 |
| QEGDTDAGLKESPLQTPT | 47 | 63 | 39 | 40 |
| GDTDAGLKESPLQTPTED | 52 | 19 | 60 | 36 |
| TDAGLKESPLQTPTEDGS | 44 | 15 | 56 | 37 |
| AGLKESPLQTPTEDGSEE | 63 | 54 | 100 | 33 |
| LKESPLQTPTEDGSEEPG | 52 | 41 | 100 | 40 |
| ESPLQTPTEDGSEEPGSE | 50 | 39 | 121 | 36 |
| PLQTPTEDGSEEPGSETS | 48 | 54 | 217 | 38 |
| QTPTEDGSEEPGSETSDA | 50 | 35 | 71 | 39 |
| PTEDGSEEPGSETSDAKS | 85 | 32 | 63 | 29 |
| EDGSEEPGSETSDAKSTP | 37 | 53 | 51 | 25 |
| GSEEPGSETSDAKSTPTA | 37 | 65 | 62 | 34 |
| EEPGSETSDAKSTPTAED | 41 | 357 | 83 | 36 |
| PGSETSDAKSTPTAEDVT | 37 | 2766 | 319 | 34 |
| SETSDAKSTPTAEDVTAP | 27 | 2828 | 67 | 41 |
| TSDAKSTPTAEDVTAPLV | 56 | 2907 | 70 | 30 |
| DAKSTPTAEDVTAPLVDE | 39 | 2911 | 98 | 27 |
| KSTPTAEDVTAPLVDEGA | 38 | 2913 | 111 | 20 |
| TPTAEDVTAPLVDEGAPG | 59 | 113 | 51 | 37 |
| TAEDVTAPLVDEGAPGKQ | 51 | 30 | 84 | 34 |
| EDVTAPLVDEGAPGKQAA | 48 | 32 | 75 | 38 |
| VTAPLVDEGAPGKQAAAQ | 56 | 41 | 94 | 37 |
| APLVDEGAPGKQAAAQPH | 50 | 47 | 69 | 44 |
| LVDEGAPGKQAAAQPHTE | 47 | 41 | 247 | 32 |
| DEGAPGKQAAAQPHTEIP | 50 | 39 | 118 | 33 |
| GAPGKQAAAQPHTEIPEG | 40 | 40 | 89 | 33 |
| PGKQAAAQPHTEIPEGTT | 37 | 36 | 61 | 29 |
| KQAAAQPHTEIPEGTTAE | 44 | 50 | 48 | 26 |
| AAAQPHTEIPEGTTAEEA | 55 | 56 | 84 | 38 |
| AQPHTEIPEGTTAEEAGI | 54 | 61 | 80 | 36 |
| PHTEIPEGTTAEEAGIGD | 51 | 67 | 117 | 37 |
| TEIPEGTTAEEAGIGDTP | 51 | 84 | 59 | 31 |
| IPEGTTAEEAGIGDTPSL | 50 | 101 | 83 | 31 |
| EGTTAEEAGIGDTPSLED | 57 | 79 | 64 | 24 |
| TTAEEAGIGDTPSLEDEA | 55 | 58 | 57 | 30 |
| AEEAGIGDTPSLEDEAAG | 79 | 59 | 81 | 37 |
| EAGIGDTPSLEDEAAGHV | 49 | 62 | 75 | 46 |
| GIGDTPSLEDEAAGHVTQ | 46 | 32 | 75 | 43 |
| GDTPSLEDEAAGHVTQAR | 47 | 32 | 72 | 34 |
| TPSLEDEAAGHVTQARMV | 52 | 35 | 55 | 40 |
| SLEDEAAGHVTQARMVSK | 76 | 66 | 82 | 79 |
| EDEAAGHVTQARMVSKSK | 44 | 46 | 54 | 49 |
| EAAGHVTQARMVSKSKDG | 56 | 55 | 73 | 67 |
| AGHVTQARMVSKSKDGTG | 42 | 36 | 62 | 58 |
| HVTQARMVSKSKDGTGSD | 38 | 41 | 42 | 39 |
| TQARMVSKSKDGTGSDDK | 45 | 56 | 43 | 32 |
| ARMVSKSKDGTGSDDKKA | 49 | 67 | 53 | 44 |
| MVSKSKDGTGSDDKKAKG | 43 | 54 | 44 | 47 |
| SKSKDGTGSDDKKAKGAD | 60 | 63 | 53 | 44 |
| SKDGTGSDDKKAKGADGK | 49 | 73 | 48 | 46 |
| DGTGSDDKKAKGADGKTK | 53 | 70 | 56 | 40 |
| TGSDDKKAKGADGKTKIA | 58 | 58 | 51 | 50 |
| SDDKKAKGADGKTKIATP | 76 | 90 | 63 | 58 |
| DKKAKGADGKTKIATPRG | 46 | 61 | 58 | 50 |
| KAKGADGKTKIATPRGAA | 52 | 61 | 58 | 39 |
| KGADGKTKIATPRGAAPP | 56 | 56 | 64 | 64 |
| ADGKTKIATPRGAAPPGQ | 68 | 75 | 63 | 47 |
| GKTKIATPRGAAPPGQKG | 66 | 45 | 64 | 67 |
| TKIATPRGAAPPGQKGQA | 48 | 55 | 58 | 60 |
| IATPRGAAPPGQKGQANA | 51 | 44 | 64 | 61 |
| TPRGAAPPGQKGQANATR | 52 | 50 | 63 | 55 |
| RGAAPPGQKGQANATRIP | 50 | 53 | 40 | 54 |
| AAPPGQKGQANATRIPAK | 53 | 58 | 56 | 63 |
| PPGQKGQANATRIPAKTP | 65 | 72 | 73 | 63 |
| GQKGQANATRIPAKTPPA | 59 | 72 | 41 | 50 |
| KGQANATRIPAKTPPAPK | 60 | 66 | 61 | 63 |
| QANATRIPAKTPPAPKTP | 56 | 82 | 65 | 51 |
| NATRIPAKTPPAPKTPPS | 54 | 67 | 53 | 59 |
| TRIPAKTPPAPKTPPSSG | 61 | 47 | 63 | 36 |
| IPAKTPPAPKTPPSSGEP | 47 | 28 | 100 | 48 |
| AKTPPAPKTPPSSGEPPK | 60 | 31 | 70 | 62 |
| TPPAPKTPPSSGEPPKSG | 57 | 45 | 96 | 50 |
| PAPKTPPSSGEPPKSGDR | 52 | 49 | 54 | 49 |
| PKTPPSSGEPPKSGDRSG | 42 | 54 | 62 | 59 |
| TPPSSGEPPKSGDRSGYS | 46 | 47 | 57 | 57 |
| PSSGEPPKSGDRSGYSSP | 44 | 33 | 80 | 54 |
| SGEPPKSGDRSGYSSPGS | 49 | 51 | 52 | 44 |
| EPPKSGDRSGYSSPGSPG | 38 | 19 | 40 | 40 |
| PKSGDRSGYSSPGSPGTP | 33 | 32 | 37 | 39 |
| SGDRSGYSSPGSPGTPGS | 37 | 54 | 41 | 33 |
| DRSGYSSPGSPGTPGSRS | 46 | 57 | 38 | 36 |
| SGYSSPGSPGTPGSRSRT | 57 | 57 | 46 | 38 |
| YSSPGSPGTPGSRSRTPS | 46 | 68 | 42 | 42 |
| SPGSPGTPGSRSRTPSLP | 52 | 47 | 45 | 42 |
| GSPGTPGSRSRTPSLPTP | 48 | 52 | 45 | 13 |
| PGTPGSRSRTPSLPTPPT | 63 | 36 | 62 | 50 |
| TPGSRSRTPSLPTPPTRE | 62 | 27 | 115 | 52 |
| GSRSRTPSLPTPPTREPK | 54 | 40 | 50 | 70 |
| RSRTPSLPTPPTREPKKV | 56 | 56 | 72 | 61 |
| RTPSLPTPPTREPKKVAV | 52 | 86 | 57 | 86 |
| PSLPTPPTREPKKVAVVR | 51 | 39 | 59 | 66 |
| LPTPPTREPKKVAVVRTP | 65 | 73 | 61 | 71 |
| TPPTREPKKVAVVRTPPK | 54 | 44 | 67 | 65 |
| PTREPKKVAVVRTPPKSP | 47 | 41 | 66 | 64 |
| REPKKVAVVRTPPKSPSS | 51 | 29 | 44 | 68 |
| PKKVAVVRTPPKSPSSAK | 40 | 50 | 45 | 50 |
| KVAVVRTPPKSPSSAKSR | 34 | 55 | 35 | 36 |
| AVVRTPPKSPSSAKSRLQ | 46 | 49 | 44 | 35 |
| VRTPPKSPSSAKSRLQTA | 40 | 67 | 40 | 43 |
| TPPKSPSSAKSRLQTAPV | 55 | 59 | 51 | 42 |
| PKSPSSAKSRLQTAPVPM | 56 | 52 | 41 | 44 |
| SPSSAKSRLQTAPVPMPD | 44 | 56 | 51 | 45 |
| SSAKSRLQTAPVPMPDLK | 56 | 66 | 62 | 83 |
| AKSRLQTAPVPMPDLKNV | 52 | 47 | 44 | 77 |
| SRLQTAPVPMPDLKNVKS | 73 | 58 | 78 | 1119 |
| LQTAPVPMPDLKNVKSKI | 49 | 54 | 80 | 1490 |
| TAPVPMPDLKNVKSKIGS | 53 | 82 | 90 | 1902 |
| PVPMPDLKNVKSKIGSTE | 49 | 78 | 107 | 2159 |
| PMPDLKNVKSKIGSTENL | 50 | 118 | 145 | 2337 |
| PDLKNVKSKIGSTENLKH | 56 | 81 | 88 | 1483 |
| LKNVKSKIGSTENLKHQP | 51 | 55 | 59 | 51 |
| NVKSKIGSTENLKHQPGG | 46 | 39 | 32 | 33 |
| KSKIGSTENLKHQPGGGK | 44 | 47 | 72 | 42 |
| KIGSTENLKHQPGGGKVQ | 53 | 58 | 109 | 53 |
| GSTENLKHQPGGGKVQII | 54 | 53 | 40 | 38 |
| TENLKHQPGGGKVQIINK | 51 | 57 | 33 | 43 |
| NLKHQPGGGKVQIINKKL | 53 | 38 | 35 | 49 |
| KHQPGGGKVQIINKKLDL | 55 | 46 | 59 | 42 |
| QPGGGKVQIINKKLDLSN | 59 | 53 | 49 | 59 |
| GGGKVQIINKKLDLSNVQ | 52 | 41 | 57 | 50 |
| GKVQIINKKLDLSNVQSK | 63 | 56 | 55 | 76 |
| VQIINKKLDLSNVQSKCG | 64 | 44 | 69 | 181 |
| IINKKLDLSNVQSKCGSK | 64 | 40 | 61 | 76 |
| NKKLDLSNVQSKCGSKDN | 59 | 55 | 68 | 88 |
| KLDLSNVQSKCGSKDNIK | 45 | 50 | 68 | 83 |
| DLSNVQSKCGSKDNIKHV | 44 | 71 | 51 | 82 |
| SNVQSKCGSKDNIKHVPG | 43 | 57 | 67 | 79 |
| VQSKCGSKDNIKHVPGGG | 45 | 46 | 62 | 51 |
| SKCGSKDNIKHVPGGGSV | 43 | 32 | 40 | 40 |
| CGSKDNIKHVPGGGSVQI | 23 | 41 | 43 | 39 |
| SKDNIKHVPGGGSVQIVY | 42 | 51 | 104 | 33 |
| DNIKHVPGGGSVQIVYKP | 55 | 51 | 31 | 31 |
| IKHVPGGGSVQIVYKPVD | 64 | 56 | 39 | 40 |
| HVPGGGSVQIVYKPVDLS | 59 | 47 | 43 | 40 |
| PGGGSVQIVYKPVDLSKV | 71 | 46 | 43 | 51 |
| GGSVQIVYKPVDLSKVTS | 57 | 42 | 61 | 60 |
| SVQIVYKPVDLSKVTSKC | 55 | 46 | 71 | 50 |
| QIVYKPVDLSKVTSKCGS | 61 | 53 | 78 | 62 |
| VYKPVDLSKVTSKCGSLG | 57 | 40 | 114 | 58 |
| KPVDLSKVTSKCGSLGNI | 65 | 64 | 48 | 62 |
| VDLSKVTSKCGSLGNIHH | 87 | 58 | 65 | 53 |
| LSKVTSKCGSLGNIHHKP | 60 | 64 | 42 | 64 |
| KVTSKCGSLGNIHHKPGG | 63 | 47 | 50 | 41 |
| TSKCGSLGNIHHKPGGGQ | 41 | 39 | 72 | 51 |
| KCGSLGNIHHKPGGGQVE | 42 | 43 | 103 | 42 |
| GSLGNIHHKPGGGQVEVK | 39 | 30 | 54 | 33 |
| LGNIHHKPGGGQVEVKSE | 26 | 56 | 60 | 41 |
| NIHHKPGGGQVEVKSEKL | 41 | 52 | 48 | 38 |
| HHKPGGGQVEVKSEKLDF | 36 | 51 | 40 | 35 |
| KPGGGQVEVKSEKLDFKD | 44 | 56 | 43 | 41 |
| GGGQVEVKSEKLDFKDRV | 39 | 48 | 56 | 43 |
| GQVEVKSEKLDFKDRVQS | 46 | 38 | 54 | 41 |
| VEVKSEKLDFKDRVQSKI | 60 | 54 | 67 | 58 |
| VKSEKLDFKDRVQSKIGS | 60 | 58 | 142 | 60 |
| SEKLDFKDRVQSKIGSLD | 48 | 40 | 55 | 42 |
| KLDFKDRVQSKIGSLDNI | 48 | 33 | 51 | 51 |
| DFKDRVQSKIGSLDNITH | 50 | 30 | 83 | 43 |
| KDRVQSKIGSLDNITHVP | 51 | 62 | 60 | 55 |
| RVQSKIGSLDNITHVPGG | 51 | 38 | 77 | 61 |
| QSKIGSLDNITHVPGGGN | 49 | 29 | 101 | 43 |
| KIGSLDNITHVPGGGNKK | 30 | 52 | 56 | 67 |
| GSLDNITHVPGGGNKKIE | 54 | 61 | 90 | 63 |
| LDNITHVPGGGNKKIETH | 55 | 45 | 63 | 48 |
| NITHVPGGGNKKIETHKL | 16 | 40 | 42 | 45 |
| THVPGGGNKKIETHKLTF | 22 | 51 | 41 | 41 |
| VPGGGNKKIETHKLTFRE | 51 | 30 | 40 | 39 |
| GGGNKKIETHKLTFRENA | 51 | 47 | 45 | 43 |
| GNKKIETHKLTFRENAKA | 49 | 49 | 54 | 45 |
| KKIETHKLTFRENAKAKT | 42 | 52 | 53 | 47 |
| IETHKLTFRENAKAKTDH | 55 | 55 | 67 | 46 |
| THKLTFRENAKAKTDHGA | 48 | 51 | 65 | 50 |
| KLTFRENAKAKTDHGAEI | 25 | 54 | 62 | 42 |
| TFRENAKAKTDHGAEIVY | 36 | 40 | 59 | 51 |
| RENAKAKTDHGAEIVYKS | 33 | 54 | 59 | 52 |
| NAKAKTDHGAEIVYKSPV | 35 | 50 | 52 | 42 |
| KAKTDHGAEIVYKSPVVS | 33 | 30 | 58 | 40 |
| KTDHGAEIVYKSPVVSGD | 36 | 44 | 78 | 42 |
| DHGAEIVYKSPVVSGDTS | 23 | 35 | 160 | 34 |
| GAEIVYKSPVVSGDTSPR | 39 | 53 | 129 | 53 |
| EIVYKSPVVSGDTSPRHL | 21 | 43 | 72 | 36 |
| VYKSPVVSGDTSPRHLSN | 21 | 43 | 78 | 46 |
| KSPVVSGDTSPRHLSNVS | 23 | 42 | 45 | 33 |
| PVVSGDTSPRHLSNVSST | 49 | 56 | 101 | 43 |
| VSGDTSPRHLSNVSSTGS | 55 | 53 | 143 | 51 |
| GDTSPRHLSNVSSTGSID | 49 | 45 | 91 | 41 |
| TSPRHLSNVSSTGSIDMV | 46 | 36 | 60 | 37 |
| PRHLSNVSSTGSIDMVDS | 42 | 51 | 78 | 43 |
| HLSNVSSTGSIDMVDSPQ | 40 | 40 | 66 | 37 |
| SNVSSTGSIDMVDSPQLA | 39 | 48 | 52 | 34 |
| VSSTGSIDMVDSPQLATL | 26 | 38 | 56 | 31 |
| STGSIDMVDSPQLATLAD | 26 | 49 | 43 | 33 |
| GSIDMVDSPQLATLADEV | 26 | 33 | 149 | 39 |
| IDMVDSPQLATLADEVSA | 33 | 32 | 372 | 39 |
| MVDSPQLATLADEVSASL | 35 | 34 | 2916 | 34 |
| DSPQLATLADEVSASLAK | 41 | 24 | 2947 | 43 |
| PQLATLADEVSASLAKQG | 35 | 20 | 2942 | 39 |
