# Supplementary material for: A Novel Tau Antibody Detecting the First Amino-Terminal Insert Reveals Conformational Differences Among Tau Isoforms
Source: Front Mol Biosci. 2020 Mar 31;7:48. doi: 10.3389/fmolb.2020.00048 (PMC7136581; doi:10.3389/fmolb.2020.00048)
Supplement: Supplementary file 2 [file Table_2.docx]

**Table S2. Linear phosphorylated peptide sequences used for epitope mapping and raw data of signal intensities for each of the four monoclonal antibodies towards linear peptides covering the amino acid sequence of human Tau2N4R.** Each linear peptide was 18 amino acids long with an overlap of 16 amino acids with the former linear peptide. Phosphorylation of peptides was designed based on possible phosphosites as described in Sergeant et al. (Sergeant et al., 2008). Phosphorylated threonine (pThr) is denoted with “4”, phosphorylated serine (pSer) is denoted with “5” and phosphorylated tyrosine (pTyr) is denoted with “7”.

| **Linear phosphorylated peptide sequence** | **15A10** | **18F12** | **20G10** | **16B12** |
| --- | --- | --- | --- | --- |
| MAEPRQEFEVMEDHAG47 | 78 | 30 | 70 | 24 |
| EPRQEFEVMEDHAG47GL | 58 | 28 | 78 | 31 |
| RQEFEVMEDHAG47GLGD | 54 | 28 | 73 | 29 |
| EFEVMEDHAG47GLGDRK | 32 | 29 | 92 | 30 |
| EVMEDHAG47GLGDRKDQ | 49 | 47 | 60 | 39 |
| MEDHAG47GLGDRKDQGG | 57 | 37 | 55 | 21 |
| DHAG47GLGDRKDQGG7T | 53 | 43 | 59 | 33 |
| AG47GLGDRKDQGG7TMH | 48 | 59 | 63 | 30 |
| 47GLGDRKDQGG7TMHQD | 71 | 41 | 70 | 38 |
| GLGDRKDQGG7TMHQDQE | 50 | 42 | 78 | 34 |
| GDRKDQGG7TMHQDQEGD | 47 | 24 | 54 | 14 |
| RKDQGG7TMHQDQEGD4D | 61 | 23 | 58 | 36 |
| DQGG7TMHQDQEGD4DAG | 47 | 25 | 51 | 35 |
| GG7TMHQDQEGD4DAGLK | 60 | 41 | 65 | 19 |
| 7TMHQDQEGD4DAGLKE5 | 46 | 49 | 68 | 32 |
| MHQDQEGD4DAGLKE5PL | 41 | 45 | 42 | 23 |
| QDQEGD4DAGLKE5PLQ4 | 40 | 46 | 44 | 29 |
| QEGD4DAGLKE5PLQ4P4 | 68 | 39 | 57 | 45 |
| GD4DAGLKE5PLQ4P4ED | 60 | 38 | 61 | 26 |
| 4DAGLKE5PLQ4P4EDG5 | 62 | 67 | 73 | 31 |
| AGLKE5PLQ4P4EDG5EE | 53 | 36 | 66 | 43 |
| LKE5PLQ4P4EDG5EEPG | 47 | 29 | 128 | 21 |
| E5PLQ4P4EDG5EEPGSE | 73 | 38 | 74 | 33 |
| PLQ4P4EDG5EEPGSETS | 64 | 42 | 67 | 15 |
| Q4P4EDG5EEPGSETSDA | 41 | 34 | 55 | 15 |
| P4EDG5EEPGSETSDAK5 | 60 | 43 | 105 | 22 |
| EDG5EEPGSETSDAK54P | 49 | 29 | 54 | 22 |
| G5EEPGSETSDAK54P4A | 58 | 45 | 88 | 33 |
| EEPGSETSDAK54P4AED | 63 | 47 | 119 | 16 |
| PGSETSDAK54P4AEDVT | 55 | 39 | 196 | 35 |
| SETSDAK54P4AEDVTAP | 49 | 41 | 46 | 35 |
| TSDAK54P4AEDVTAPLV | 53 | 36 | 55 | 31 |
| DAK54P4AEDVTAPLVDE | 62 | 41 | 74 | 41 |
| K54P4AEDVTAPLVDEGA | 61 | 39 | 117 | 37 |
| 4P4AEDVTAPLVDEGAPG | 39 | 33 | 258 | 17 |
| 4AEDVTAPLVDEGAPGKQ | 64 | 29 | 55 | 22 |
| EDVTAPLVDEGAPGKQAA | 39 | 41 | 83 | 36 |
| VTAPLVDEGAPGKQAAAQ | 42 | 32 | 58 | 26 |
| APLVDEGAPGKQAAAQPH | 48 | 25 | 45 | 17 |
| LVDEGAPGKQAAAQPH4E | 55 | 39 | 212 | 28 |
| DEGAPGKQAAAQPH4EIP | 41 | 27 | 33 | 28 |
| GAPGKQAAAQPH4EIPEG | 59 | 45 | 53 | 39 |
| PGKQAAAQPH4EIPEG44 | 69 | 41 | 61 | 34 |
| KQAAAQPH4EIPEG44AE | 61 | 43 | 56 | 29 |
| AAAQPH4EIPEG44AEEA | 73 | 34 | 88 | 46 |
| AQPH4EIPEG44AEEAGI | 74 | 34 | 86 | 41 |
| PH4EIPEG44AEEAGIGD | 72 | 41 | 90 | 51 |
| 4EIPEG44AEEAGIGDTP | 65 | 47 | 65 | 37 |
| IPEG44AEEAGIGDTP5L | 63 | 39 | 121 | 19 |
| EG44AEEAGIGDTP5LED | 73 | 10 | 64 | 38 |
| 44AEEAGIGDTP5LEDEA | 61 | 42 | 79 | 28 |
| AEEAGIGDTP5LEDEAAG | 96 | 49 | 65 | 29 |
| EAGIGDTP5LEDEAAGHV | 54 | 45 | 52 | 41 |
| GIGDTP5LEDEAAGHV4Q | 70 | 37 | 61 | 27 |
| GDTP5LEDEAAGHV4QAR | 92 | 29 | 44 | 27 |
| TP5LEDEAAGHV4QARMV | 53 | 33 | 55 | 30 |
| 5LEDEAAGHV4QARMVSK | 41 | 32 | 33 | 39 |
| EDEAAGHV4QARMVSK5K | 41 | 38 | 41 | 36 |
| EAAGHV4QARMVSK5KDG | 55 | 32 | 48 | 35 |
| AGHV4QARMVSK5KDGTG | 45 | 31 | 54 | 36 |
| HV4QARMVSK5KDGTGSD | 53 | 37 | 60 | 14 |
| 4QARMVSK5KDGTGSDDK | 45 | 23 | 89 | 28 |
| ARMVSK5KDGTGSDDKKA | 97 | 26 | 51 | 25 |
| MVSK5KDGTGSDDKKAKG | 62 | 28 | 59 | 34 |
| SK5KDGTGSDDKKAKGAD | 42 | 27 | 52 | 36 |
| 5KDGTGSDDKKAKGADGK | 96 | 46 | 64 | 62 |
| DGTGSDDKKAKGADGK4K | 54 | 49 | 49 | 32 |
| TGSDDKKAKGADGK4KIA | 43 | 44 | 46 | 45 |
| SDDKKAKGADGK4KIA4P | 47 | 32 | 31 | 29 |
| DKKAKGADGK4KIA4PRG | 58 | 73 | 40 | 44 |
| KAKGADGK4KIA4PRGAA | 68 | 46 | 44 | 34 |
| KGADGK4KIA4PRGAAPP | 56 | 48 | 52 | 35 |
| ADGK4KIA4PRGAAPPGQ | 104 | 29 | 181 | 34 |
| GK4KIA4PRGAAPPGQKG | 71 | 34 | 89 | 50 |
| 4KIA4PRGAAPPGQKGQA | 54 | 56 | 66 | 51 |
| IA4PRGAAPPGQKGQANA | 50 | 44 | 131 | 34 |
| 4PRGAAPPGQKGQANA4R | 58 | 39 | 137 | 33 |
| RGAAPPGQKGQANA4RIP | 62 | 43 | 63 | 34 |
| AAPPGQKGQANA4RIPAK | 57 | 56 | 75 | 39 |
| PPGQKGQANA4RIPAK4P | 53 | 14 | 90 | 26 |
| GQKGQANA4RIPAK4PPA | 53 | 34 | 46 | 30 |
| KGQANA4RIPAK4PPAPK | 59 | 33 | 62 | 27 |
| QANA4RIPAK4PPAPK4P | 86 | 24 | 62 | 40 |
| NA4RIPAK4PPAPK4PP5 | 38 | 32 | 98 | 19 |
| 4RIPAK4PPAPK4PP5SG | 60 | 35 | 127 | 36 |
| IPAK4PPAPK4PP5SGEP | 62 | 32 | 203 | 36 |
| AK4PPAPK4PP5SGEPPK | 46 | 33 | 43 | 45 |
| 4PPAPK4PP5SGEPPK5G | 125 | 41 | 84 | 21 |
| PAPK4PP5SGEPPK5GDR | 34 | 37 | 49 | 38 |
| PK4PP5SGEPPK5GDRSG | 287 | 19 | 68 | 27 |
| 4PP5SGEPPK5GDRSG75 | 51 | 39 | 74 | 46 |
| P5SGEPPK5GDRSG755P | 67 | 39 | 124 | 32 |
| SGEPPK5GDRSG755PG5 | 69 | 38 | 92 | 60 |
| EPPK5GDRSG755PG5PG | 969 | 41 | 73 | 33 |
| PK5GDRSG755PG5PG4P | 2695 | 33 | 60 | 33 |
| 5GDRSG755PG5PG4PG5 | 934 | 35 | 144 | 40 |
| DRSG755PG5PG4PG5R5 | 2771 | 36 | 101 | 34 |
| SG755PG5PG4PG5R5R4 | 227 | 26 | 96 | 24 |
| 755PG5PG4PG5R5R4P5 | 2910 | 60 | 97 | 35 |
| 5PG5PG4PG5R5R4P5LP | 2946 | 49 | 157 | 27 |
| G5PG4PG5R5R4P5LP4P | 2896 | 39 | 148 | 46 |
| PG4PG5R5R4P5LP4PP4 | 78 | 41 | 119 | 32 |
| 4PG5R5R4P5LP4PP4RE | 77 | 43 | 156 | 32 |
| G5R5R4P5LP4PP4REPK | 56 | 25 | 154 | 34 |
| R5R4P5LP4PP4REPKKV | 74 | 37 | 162 | 43 |
| R4P5LP4PP4REPKKVAV | 105 | 56 | 155 | 59 |
| P5LP4PP4REPKKVAVVR | 79 | 46 | 68 | 50 |
| LP4PP4REPKKVAVVR4P | 62 | 29 | 197 | 43 |
| 4PP4REPKKVAVVR4PPK | 56 | 46 | 52 | 36 |
| P4REPKKVAVVR4PPK5P | 84 | 42 | 96 | 46 |
| REPKKVAVVR4PPK5P55 | 48 | 36 | 44 | 43 |
| PKKVAVVR4PPK5P55AK | 59 | 54 | 91 | 57 |
| KVAVVR4PPK5P55AK5R | 130 | 41 | 68 | 42 |
| AVVR4PPK5P55AK5RLQ | 68 | 31 | 202 | 160 |
| VR4PPK5P55AK5RLQ4A | 67 | 28 | 98 | 41 |
| 4PPK5P55AK5RLQ4APV | 61 | 25 | 167 | 32 |
| PK5P55AK5RLQ4APVPM | 61 | 29 | 231 | 24 |
| 5P55AK5RLQ4APVPMPD | 27 | 28 | 106 | 25 |
| 55AK5RLQ4APVPMPDLK | 52 | 38 | 61 | 42 |
| AK5RLQ4APVPMPDLKNV | 45 | 61 | 113 | 36 |
| 5RLQ4APVPMPDLKNVK5 | 45 | 34 | 120 | 149 |
| LQ4APVPMPDLKNVK5KI | 59 | 32 | 62 | 597 |
| 4APVPMPDLKNVK5KIG5 | 75 | 60 | 80 | 1321 |
| PVPMPDLKNVK5KIG54E | 56 | 39 | 153 | 342 |
| PMPDLKNVK5KIG54ENL | 36 | 32 | 79 | 250 |
| PDLKNVK5KIG54ENLKH | 58 | 42 | 65 | 323 |
| LKNVK5KIG54ENLKHQP | 85 | 18 | 53 | 28 |
| NVK5KIG54ENLKHQPGG | 50 | 39 | 51 | 37 |
| K5KIG54ENLKHQPGGGK | 58 | 48 | 102 | 39 |
| KIG54ENLKHQPGGGKVQ | 72 | 47 | 69 | 58 |
| G54ENLKHQPGGGKVQII | 78 | 30 | 48 | 39 |
| 4ENLKHQPGGGKVQIINK | 129 | 28 | 55 | 42 |
| NLKHQPGGGKVQIINKKL | 66 | 76 | 47 | 49 |
| KHQPGGGKVQIINKKLDL | 59 | 37 | 54 | 47 |
| QPGGGKVQIINKKLDL5N | 54 | 50 | 52 | 49 |
| GGGKVQIINKKLDL5NVQ | 55 | 46 | 52 | 43 |
| GKVQIINKKLDL5NVQ5K | 54 | 37 | 70 | 30 |
| VQIINKKLDL5NVQ5KCG | 50 | 24 | 61 | 43 |
| IINKKLDL5NVQ5KCG5K | 47 | 25 | 83 | 27 |
| NKKLDL5NVQ5KCG5KDN | 45 | 33 | 42 | 34 |
| KLDL5NVQ5KCG5KDNIK | 66 | 25 | 54 | 14 |
| DL5NVQ5KCG5KDNIKHV | 42 | 27 | 37 | 13 |
| 5NVQ5KCG5KDNIKHVPG | 63 | 40 | 57 | 21 |
| VQ5KCG5KDNIKHVPGGG | 80 | 35 | 72 | 48 |
| 5KCG5KDNIKHVPGGG5V | 60 | 32 | 72 | 37 |
| CG5KDNIKHVPGGG5VQI | 70 | 42 | 76 | 45 |
| 5KDNIKHVPGGG5VQIVY | 59 | 38 | 62 | 43 |
| DNIKHVPGGG5VQIVYKP | 66 | 39 | 83 | 35 |
| IKHVPGGG5VQIVYKPVD | 70 | 42 | 91 | 25 |
| HVPGGG5VQIVYKPVDL5 | 52 | 35 | 65 | 31 |
| PGGG5VQIVYKPVDL5KV | 36 | 21 | 62 | 22 |
| GG5VQIVYKPVDL5KVT5 | 70 | 36 | 73 | 37 |
| 5VQIVYKPVDL5KVT5KC | 51 | 25 | 82 | 29 |
| QIVYKPVDL5KVT5KCG5 | 61 | 11 | 132 | 30 |
| VYKPVDL5KVT5KCG5LG | 70 | 52 | 114 | 795 |
| KPVDL5KVT5KCG5LGNI | 71 | 34 | 65 | 35 |
| VDL5KVT5KCG5LGNIHH | 108 | 32 | 78 | 17 |
| L5KVT5KCG5LGNIHHKP | 49 | 31 | 69 | 18 |
| KVT5KCG5LGNIHHKPGG | 60 | 39 | 53 | 30 |
| T5KCG5LGNIHHKPGGGQ | 69 | 22 | 90 | 26 |
| KCG5LGNIHHKPGGGQVE | 56 | 28 | 68 | 39 |
| G5LGNIHHKPGGGQVEVK | 66 | 48 | 81 | 32 |
| LGNIHHKPGGGQVEVK5E | 50 | 46 | 103 | 30 |
| NIHHKPGGGQVEVK5EKL | 48 | 33 | 55 | 25 |
| HHKPGGGQVEVK5EKLDF | 54 | 30 | 58 | 37 |
| KPGGGQVEVK5EKLDFKD | 38 | 27 | 39 | 10 |
| GGGQVEVK5EKLDFKDRV | 63 | 35 | 49 | 22 |
| GQVEVK5EKLDFKDRVQ5 | 49 | 36 | 55 | 38 |
| VEVK5EKLDFKDRVQ5KI | 52 | 39 | 54 | 40 |
| VK5EKLDFKDRVQ5KIG5 | 54 | 49 | 145 | 71 |
| 5EKLDFKDRVQ5KIG5LD | 48 | 39 | 52 | 32 |
| KLDFKDRVQ5KIG5LDNI | 53 | 38 | 72 | 19 |
| DFKDRVQ5KIG5LDNI4H | 55 | 40 | 52 | 33 |
| KDRVQ5KIG5LDNI4HVP | 79 | 39 | 66 | 35 |
| RVQ5KIG5LDNI4HVPGG | 62 | 43 | 76 | 34 |
| Q5KIG5LDNI4HVPGGGN | 32 | 29 | 41 | 30 |
| KIG5LDNI4HVPGGGNKK | 55 | 37 | 50 | 30 |
| G5LDNI4HVPGGGNKKIE | 84 | 39 | 81 | 43 |
| LDNI4HVPGGGNKKIE4H | 84 | 47 | 95 | 35 |
| NI4HVPGGGNKKIE4HKL | 52 | 41 | 69 | 38 |
| 4HVPGGGNKKIE4HKLTF | 51 | 27 | 51 | 36 |
| VPGGGNKKIE4HKLTFRE | 47 | 44 | 64 | 35 |
| GGGNKKIE4HKLTFRENA | 53 | 38 | 43 | 38 |
| GNKKIE4HKLTFRENAKA | 89 | 48 | 71 | 54 |
| KKIE4HKLTFRENAKAK4 | 162 | 39 | 85 | 34 |
| IE4HKLTFRENAKAK4DH | 61 | 35 | 194 | 38 |
| 4HKLTFRENAKAK4DHGA | 82 | 39 | 94 | 36 |
| KLTFRENAKAK4DHGAEI | 57 | 33 | 97 | 42 |
| TFRENAKAK4DHGAEIV7 | 52 | 36 | 214 | 30 |
| RENAKAK4DHGAEIV7K5 | 49 | 41 | 50 | 30 |
| NAKAK4DHGAEIV7K5PV | 70 | 48 | 89 | 41 |
| KAK4DHGAEIV7K5PVV5 | 69 | 20 | 87 | 28 |
| K4DHGAEIV7K5PVV5GD | 58 | 33 | 104 | 36 |
| DHGAEIV7K5PVV5GD45 | 81 | 58 | 80 | 42 |
| GAEIV7K5PVV5GD45PR | 57 | 29 | 187 | 30 |
| EIV7K5PVV5GD45PRHL | 53 | 42 | 61 | 26 |
| V7K5PVV5GD45PRHL5N | 49 | 35 | 44 | 43 |
| K5PVV5GD45PRHL5NV5 | 86 | 20 | 236 | 43 |
| PVV5GD45PRHL5NV554 | 40 | 21 | 109 | 29 |
| V5GD45PRHL5NV554G5 | 56 | 34 | 351 | 38 |
| GD45PRHL5NV554G5ID | 47 | 27 | 79 | 33 |
| 45PRHL5NV554G5IDMV | 65 | 53 | 93 | 40 |
| PRHL5NV554G5IDMVD5 | 81 | 39 | 77 | 44 |
| HL5NV554G5IDMVD5PQ | 56 | 44 | 61 | 30 |
| 5NV554G5IDMVD5PQLA | 46 | 44 | 74 | 35 |
| V554G5IDMVD5PQLA4L | 55 | 41 | 60 | 37 |
| 54G5IDMVD5PQLA4LAD | 57 | 36 | 60 | 35 |
| G5IDMVD5PQLA4LADEV | 67 | 44 | 75 | 20 |
| IDMVD5PQLA4LADEV5A | 60 | 121 | 117 | 39 |
| MVD5PQLA4LADEV5A5L | 56 | 43 | 88 | 42 |
| D5PQLA4LADEV5A5LAK | 82 | 47 | 299 | 30 |
| PQLA4LADEV5A5LAKQG | 49 | 44 | 926 | 33 |
